# Training and Validation of Deep Learning-Based Auto-Segmentation Models for Lung Stereotactic Ablative Radiotherapy Using Retrospective Radiotherapy Planning Contours

**DOI:** 10.3389/fonc.2021.626499

**Published:** 2021-06-07

**Authors:** Jordan Wong, Vicky Huang, Joshua A. Giambattista, Tony Teke, Carter Kolbeck, Jonathan Giambattista, Siavash Atrchian

**Affiliations:** ^1^ Radiation Oncology, British Columbia Cancer – Vancouver, Vancouver, BC, Canada; ^2^ Medical Physics, British Columbia Cancer – Fraser Valley, Surrey, BC, Canada; ^3^ Radiation Oncology, Saskatchewan Cancer Agency, Regina, SK, Canada; ^4^ Limbus AI Inc, Regina, SK, Canada; ^5^ Medical Physics/Radiation Oncology, British Columbia Cancer – Kelowna, Kelowna, BC, Canada

**Keywords:** machine learning, radiotherapy, radiotherapy plan, computer-assist, stereotactic ablative body radiation

## Abstract

**Purpose:**

Deep learning-based auto-segmented contour (DC) models require high quality data for their development, and previous studies have typically used prospectively produced contours, which can be resource intensive and time consuming to obtain. The aim of this study was to investigate the feasibility of using retrospective peer-reviewed radiotherapy planning contours in the training and evaluation of DC models for lung stereotactic ablative radiotherapy (SABR).

**Methods:**

Using commercial deep learning-based auto-segmentation software, DC models for lung SABR organs at risk (OAR) and gross tumor volume (GTV) were trained using a deep convolutional neural network and a median of 105 contours per structure model obtained from 160 publicly available CT scans and 50 peer-reviewed SABR planning 4D-CT scans from center A. DCs were generated for 50 additional planning CT scans from center A and 50 from center B, and compared with the clinical contours (CC) using the Dice Similarity Coefficient (DSC) and 95% Hausdorff distance (HD).

**Results:**

Comparing DCs to CCs, the mean DSC and 95% HD were 0.93 and 2.85mm for aorta, 0.81 and 3.32mm for esophagus, 0.95 and 5.09mm for heart, 0.98 and 2.99mm for bilateral lung, 0.52 and 7.08mm for bilateral brachial plexus, 0.82 and 4.23mm for proximal bronchial tree, 0.90 and 1.62mm for spinal cord, 0.91 and 2.27mm for trachea, and 0.71 and 5.23mm for GTV. DC to CC comparisons of center A and center B were similar for all OAR structures.

**Conclusions:**

The DCs developed with retrospective peer-reviewed treatment contours approximated CCs for the majority of OARs, including on an external dataset. DCs for structures with more variability tended to be less accurate and likely require using a larger number of training cases or novel training approaches to improve performance. Developing DC models from existing radiotherapy planning contours appears feasible and warrants further clinical workflow testing.

## Introduction

Stereotactic ablative radiotherapy (SABR) is an effective treatment for both primary lung cancers and oligometastatic lesions in the lung (1, 2). This technique uses high doses per fraction and small margins, so accurate contouring of organs at risk (OARs) and gross tumor volumes (GTVs) is particularly important.

The use of auto-segmentation can ease workflow pressure and improve treatment consistency by streamlining manual contouring tasks (3, 4) and potentially reducing inter-observer variability (IOV). While deep learning-based auto-segmented contours (DCs) have been shown to closely approximate manual contours (5) and have improved results over atlas-based contours (6), they are not yet widely used in clinical practice (7). The quality of different DC models can vary significantly (8, 9), so studies verifying the reliability of a specific model are needed (10).

Well-performing DC models require high quality contours for their development. A large dataset of training and validation cases is desirable to adequately expose and evaluate the model in the variety of scenarios that may be encountered during clinical use (10, 11). Manual segmentation by experts can be considered the gold standard for accurate contours, but collecting prospective expert contours for DC model development requires significant amounts of time and resources (5).

Existing radiotherapy plans of previously treated patients can be a potential alternative or supplement to prospective study contours; these are more easily obtainable but are currently uncommonly utilized for the development of DC models (6, 8, 9, 12). Manual contours from existing radiotherapy plans are approved for treatment planning, and in many cases are peer-reviewed, so they can likely be considered of sufficiently high quality for the development of auto-segmentation models, even if they may not always be as carefully generated as prospective expert study contours.

Being able to leverage existing treatment-approved radiotherapy plans could allow for expedited auto-segmentation model development, greatly expand the pool of high quality data available for model development, and facilitate the implementation of novel auto-segmentation models into the clinical workflow. However, training and validating with treatment-approved planning contours from only a single center will bring concerns of whether the resulting DC models would be applicable to other institutions or patient populations; therefore, independent evaluation is critical (10).

The purpose of this study is to investigate whether using retrospective radiotherapy planning contours in the development of auto-segmentation models is feasible, and to assess the performance of these models, including on cases from a different center. We train DCs for lung SABR OARs and GTV with the goal of showing that these auto-segmentation models are of adequate accuracy to warrant further clinical workflow testing.

## Methods

### Deep Learning-Based Auto-Segmentation Models

The commercial deep learning-based auto-segmentation software, Limbus Contour version 1.0.22, uses deep convolutional neural networks (one model per structure) based on a U-net architecture (13–15). This software was provided without cost through a research agreement. Auto-segmentation models are developed from the training dataset and implemented using TensorFlow (16). Data augmentation (flipping, brightness adjustments, elastic deformations) and regularization techniques (dropout, batch normalization) are used during training to improve model performance and prevent overfitting. Post processing of the DCs before finalized structure set creation includes 3D volumetric outlier removal, confidence score/area anomaly-based slice interpolation, statistical (outlier)-based z-plane cutoffs, and contour smoothing. Further details of model generation and optimization methods used by this commercial auto-segmentation software have not been made public by the manufacturer.

### Training Dataset

Approval for this study was obtained from our institutional research ethics board. A total of 210 contoured computed tomography (CT) scans were obtained for training of DCs; only structures already contoured on these scans were included in the training dataset as not every case had all of the structures of interest contoured (e.g., no aorta for treatment of peripheral tumor). Developed DC models and the number of training cases with that structure included: aorta (n = 34), esophagus (n = 156), heart (n = 191), left lung (n = 174), right lung (n = 177), left brachial plexus (BP; n = 58), right BP (n = 56), proximal bronchial tree (PBT; n = 88), spinal cord (n = 105), trachea (n = 143), and GTV (n = 96). The median number of contours used for training a structure model was 105 (range 34 to 191). The GTVs did not encompass all physiological tumor motions seen on a 4D CT scan, so they did not represent an internal target volume or internal GTV.

Publicly available CT scans with lung OAR and GTV contours were obtained from datasets accessible through the Cancer Imaging Archive (17). These datasets included contoured CT scans from auto-segmentation studies, proteogenomic studies, image verification studies, and conventional radiotherapy plans (18–22). One hundred and sixty publicly available CT scans containing lung OAR and/or GTV contours were utilized, and their existing contours were reviewed and edited by an external radiation oncologist according to consensus guidelines (23, 24) prior to training. The majority of these cases did not use intravenous (IV) contrast and were free-breathing image sets. Specific proportions of IV contrast use and CT image set used were not reported in these datasets. The other 50 training cases were lung SABR plans used for patient treatment at center A. Appropriate cases were identified in reverse chronological order starting from study conception and encompassed a period between January 2017 and August 2018. Planning 4D CT images were captured using a GE Healthcare Optima CT580 series scanner with the following parameters: 120kVp, 100-440mAs, 2.5 mm slice thickness, 1.270mm in-plane pixel size, and 65cm field of view. Contours from these plans were created and peer-reviewed prior to treatment by radiation oncologists at that center. After selection for use in this study, an external radiation oncologist converted the spinal cord if only a spinal canal contour was available, and extracted an aorta contour from a ‘Great Vessel’ structure. The 4D CT average image set in 46 cases was used for treatment planning and DC model training, while the free-breathing image set was used in the other 4 cases. None of these 50 cases were scanned with IV contrast.

Patient demographics, tumor characteristics, and tumor locations of the 160 publicly available cases used for training were not available. In the 50 training cases from center A, 46% were male, the median age was 76 years (range 40–93 years), and 74% of patients were being treated for a primary lung cancer as opposed to oligometastatic disease in the lung. The 54 lung nodules in these cases included 19 left upper lobe (35%), 5 left lower lobe (9%), 17 right upper lobe (31%), 3 right middle lobe (6%), and 10 right lower lobe (19%) nodules.

### Validation Dataset

DCs for lung OARs and GTV were generated on 100 planning CT scans used for lung SABR treatment. The DCs were generated in a median of 3.6 min per patient (range 1.0–4.7 min) using a MacBook Pro (2018, 2.3 GHz Intel Core i5, 16 GB 2133 MHz LPDDR3). The 100 validation cases consisted of 50 additional cases from center A and 50 from center B. Center A cases were chosen in reverse chronological order after model training was complete and contained cases treated between December 2015 and February 2019; there was no overlap with cases in the training dataset. Center B cases were treated between August 2016 to October 2018, also being selected in reverse chronological order. Center B images were captured using a GE Lightspeed RT16 using 120kVp, 400mAs, slice thickness 2.5mm, 0.9766mm in-plane pixel size, and 50cm field of view. The original manual clinical contours (CCs) on all planning scans underwent peer-review prior to treatment and after selection for use in this study, and aorta contours were extracted from the “Great Vessel” structure when necessary by an external RO; no further review or adjustments were performed. Twenty-two of the 50 cases from center A were planned on the 4D CT average image sets, while the remaining 28 cases were free-breathing image sets. The average image set was used in 49 of 50 cases from center B. One case from center A and no cases from center B had IV contrast.

In the 100 validation cases, 54% were male, the median age was 76 years (range 52–91 years), and 85% of patients were being treated for a primary lung cancer as opposed to oligometastatic disease in the lung. The 106 treated lung nodules in these cases included 28 left upper lobe (26%), 17 left lower lobe (16%), 39 right upper lobe (37%), 4 right middle lobe (4%), and 18 right lower lobe (17%) nodules.

The CCs for each validation case were compared with DCs using a software function written in Python to calculate the Dice Similarity Coefficient (DSC) and 95% Hausdorff distance (HD). DSC and 95% HD are derived from each contour 3D volume, and 3D volumes were reconstructed from output data DICOM RT-Structure Set Contour Data. DSC represents the relative overlap of segmentation volumes and can range from 0 to 1, with 0 indicating no overlap and 1 for perfect overlap. The 95% HD is the distance that represents the largest surface-to-surface separation among the closest 95% of surface points.

Nodules segmented by the GTV DC model that did not have a corresponding CC were ignored; the specificity of this model was not evaluated in this study as false positives can be easily deleted by users in clinical workflow. Only spinal canal CCs were present for most cases and were compared to spinal cord DCs. The CCs tended to only include the portion of BP, spinal cord/canal, and aorta in the areas in close proximity to the target volume so the superior and inferior borders of these DC structures were cropped to the same axial planes prior to comparison. Similar cropping was done for the inferior trachea and superior PBT DCs. Fourteen BP CCs from center A were contoured on non-consecutive slices (e.g., every 3rd or 4th slice) and excluded from the contour comparison analysis.

## Results

**Figure 1:**
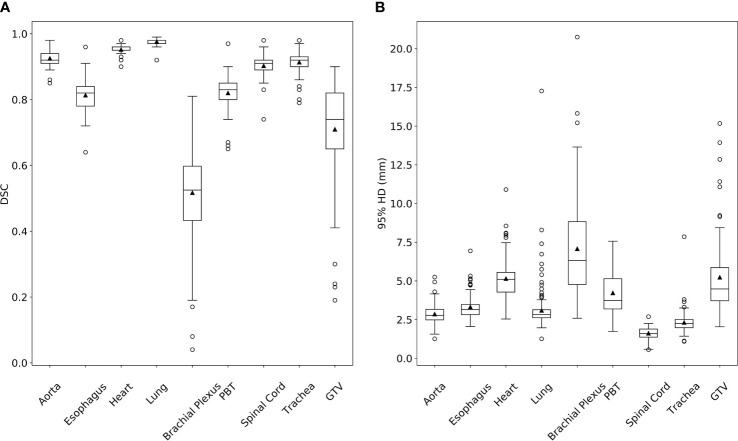
Dice Similarity Coefficient (DSC, **A**) and 95% Hausdorff distance (HD, **B**) box plots from comparing deep learning-based auto-segmented contours to clinical contours for lung stereotactic ablative radiotherapy planning structures. (PBT, proximal bronchial tree; GTV, gross tumor volume).

The DC to CC (DC-CC) DSC and 95% HD values for the validation cases are summarized in **Table 1** and presented in **Figure 1**. Example images of DCs and CCs for all structures can be found in the **Supplementary Files**.

**Table 1:** Summary of Dice Similarity Coefficient (DSC) and 95% Hausdorff distance (HD) metrics from comparing deep learning-based auto-segmented contours to clinical contours for lung stereotactic ablative radiotherapy planning structures.

Structure	N	Median DSC	Mean DSC (range)	Median 95% HD (mm)	Mean 95% HD (range; mm)
Aorta	81	0.92	0.93 (0.85–0.98)	2.77	2.85 (1.26-5.25)
Esophagus	99	0.82	0.81 (0.64–0.96)	3.15	3.32 (2.05–6.94)
Heart	100	0.95	0.95 (0.87–0.98)	4.48	5.09 (2.54–8.55)
Lung Bilateral	188	0.98	0.98 (0.92–0.99)	2.83	2.99 (1.26–6.73)
Lung Left	93	0.98	0.98 (0.92–0.99)	2.74	2.93 (1.97–6.73)
Lung Right	95	0.98	0.98 (0.96–0.99)	2.91	3.04 (1.26–5.40)
Brachial Plexus	90	0.53	0.52 (0.04–0.81)	6.3	7.08 (2.59–20.75)
Brachial Plexus Left	47	0.53	0.53 (0.17–0.81)	5.95	6.88 (2.74–15.82)
Brachial Plexus Right	43	0.52	0.5 (0.04–0.80)	6.4	7.29 (2.59–20.75)
Proximal Bronchial Tree	100	0.83	0.82 (0.65–0.97)	3.74	4.23 (1.73–7.56)
Spinal Cord	100	0.91	0.9 (0.74–0.98)	1.6	1.62 (0.56–2.69)
Trachea	100	0.92	0.91 (0.79–0.98)	2.25	2.27 (1.09–3.80)
GTV	85	0.74	0.71 (0.19–0.90)	4.48	5.23 (2.04–15.17)

(N, number of validation contours evaluated; GTV, gross tumor volume).

Twenty-one lung nodules did not have a GTV DC-CC comparison. Seven of these cases only had an internal GTV CC, which takes into account other CT image sets, so a comparison was not performed. The Limbus Contour software did not generate a DC in 11 cases. The remaining three lung nodule instances were in close proximity to another nodule, with both being included in a single GTV; these GTVs were still included in the analysis and compared to the closest matching GTV DC.

**Figure 2:**
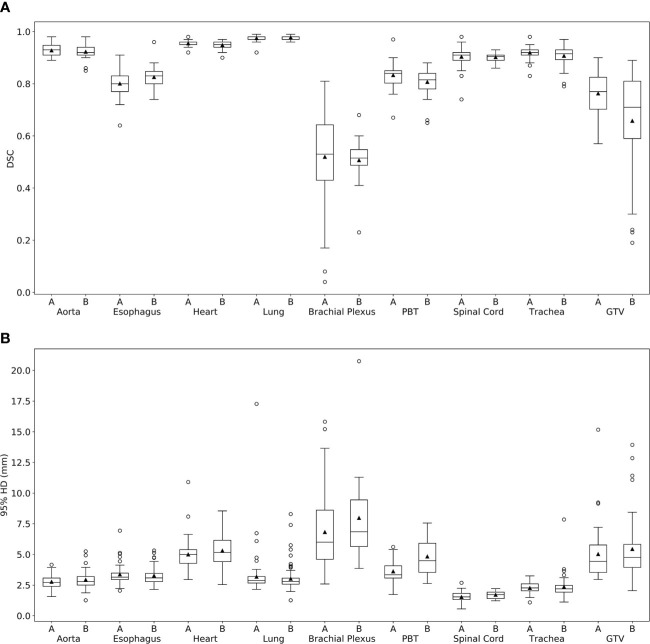
Dice Similarity Coefficient (DSC) and 95% Hausdorff distance (HD) box plots from comparing deep learning-based auto-segmented contours to clinical contours for center **(A)** and center **(B)** lung stereotactic ablative radiotherapy planning structures. (PBT, proximal bronchial tree; GTV, gross tumor volume).

Comparison metrics according to validation case center are shown in **Figure 2** and summarized in **Table 2**. Similar DC-CC values are seen between the two centers for the aorta, esophagus, heart, lungs, BP, PBT, spinal cord, and trachea. Less similarity was seen for the mean GTV DSC and 95% HD, which were 0.76 and 5.02mm for center A, and 0.66 and 5.44mm for center B.

**Table 2:** Summary of Dice Similarity Coefficient (DSC) and 95% Hausdorff distance (HD) metrics from comparing deep learning-based auto-segmented contours to clinical contours for center A and center B lung stereotactic ablative radiotherapy planning structures.

Structure	Center A N	Center A Mean DSC (range)	Center A Mean 95%HD (range; mm)	Center B N	Center B Mean DSC (range)	Center B Mean 95% HD(range; mm)
Aorta	38	0.93 (0.89–0.98)	2.77 (1.56–4.16)	43	0.92 (0.85–0.98)	2.93 (1.26-5.25)
Esophagus	49	0.80 (0.64–0.91)	3.37 (2.05–6.94)	50	0.83 (0.71–0.96)	3.27 (2.14-5.32)
Heart	50	0.95 (0.87–0.98)	4.89 (2.95–8.09)	50	0.95 (0.87–0.97)	5.30 (2.54-8.55)
Lung Left	43	0.97 (0.92–0.99)	2.97 (2.15–6.73)	50	0.98 (0.96–0.99)	2.89 (1.97-5.75)
Lung Right	45	0.97 (0.96–0.99)	3.08 (2.55–4.74)	50	0.98 (0.96–0.99)	3.00 (1.26-5.40)
Brachial Plexus	70	0.52 (0.04–0.81)	6.82 (2.59–15.82)	20	0.52 (0.23–0.68)	7.97 (3.86-20.75)
Proximal Bronchial Tree	50	0.83 (0.67–0.97)	3.62 (1.73–5.61)	50	0.81 (0.65–0.88)	4.83 (2.63-7.56)
Spinal Cord	50	0.90 (0.74–0.98)	1.53 (0.56–2.69)	50	0.90 (0.86–0.93)	1.71 (1.22-2.21)
Trachea	50	0.92 (0.83–0.98)	2.26 (1.09–3.25)	50	0.91 (0.79–0.97)	2.27 (1.12–3.80)
GTV	42	0.76 (0.57–0.90)	5.02 (2.96–15.17)	43	0.66 (0.19–0.89)	5.44 (2.04–13.93)

(N, number of validation contours evaluated; GTV, gross tumor volume).

DC-CC comparison metrics for center A free-breathing and average 4D CT scan image sets are reported in the **Supplementary Files**. Similar DSC and 95% HD values were seen for the OARs and GTV for these two groups.

## Discussion

In this study, we investigated the feasibility of developing DC models with retrospective radiotherapy planning contours. We trained DCs for lung SABR OARs and GTVs using publicly available data and existing radiotherapy planning contours from one center and evaluated their accuracy to peer-reviewed manual contours on radiotherapy plans from the same and a different center. We observed that the DCs for most OARs approximated the CCs, regardless of the center from which the validation cases were obtained. The DCs for structures that were associated with more contouring variability between cases tended to have less similarity to the CCs, but still had results comparable to other auto-segmentation studies; these structures are discussed further in subsequent paragraphs.

It is difficult to define a DSC or 95% HD threshold at which DCs are felt to be clinically useful since manual contours from different clinicians will inevitably demonstrate some amount of IOV (21, 25). Additionally, Cha et al. investigated prostate radiotherapy DCs in the clinical workflow and found that geometric indices when comparing the unedited and final contours were not strongly correlated with contouring times or reported quality scores (4). Editing inaccurate DCs may therefore still provide a workflow benefit and time savings over fully manually contoured structures (4, 6, 26), and any statistical analysis into the comparison metrics would be difficult to interpret clinically. For these reasons, we evaluate the DC model performance by comparing our DSC and 95% HD results with other auto-segmentation and IOV studies.

Previous literature that reported DSC and/or 95% HD metrics for lung SABR OARs and GTVs have assessed similar DC models (6, 8, 9, 12), investigated other auto-segmentation methods (27–30), and/or reported on the IOV of manual contours (21, 25, 28). These studies are compiled in **Table 3** alongside our results. Among the deep learning-based auto-segmentation studies, manual contours from these studies were mostly obtained through prospective contouring or from open-source diagnostic image sets that included manual contours (8, 9, 12). Only one study used prior radiotherapy planning contours for training (6), and no studies used existing radiotherapy contours for validation.

**Table 3:** Summary of Dice Similarity Coefficient (DSC) and 95% Hausdorff distance (HD) metrics for lung stereotactic ablative radiotherapy planning structures from the current study and other studies.

Structure	^a^Current Study Number of Cases	Current Study Mean DSC	Current Study Mean 95% HD (mm)	Study Number of Cases	Study DSC	Study 95% HD (mm)
Aorta	T = 34	0.93	2.85	^a,12^T = 10	0.83–0.91	1.56–2.44
	V = 81			V = 10		
Esophagus	T = 156	0.81	3.32	^b,21^N/A	0.82	3.33
	V = 99					
				^b,25^NA	0.64	–
				^a,6^T = 450	0.70	6
				V = 20		
				^c,27^T = N/A	0.49	30.6
				V = 24		
Heart	T = 191	0.95	5.09	^b,21^N/A	0.93	6.42
	V = 100					
				^b,25^NA	0.92	–
				^a,6^T = 450	0.90	13
				V = 20		
				^c,27^T = N/A	0.78	31.2
				V = 24		
Lung Left	T = 174	0.98	2.93	^b,21^N/A	0.96	5.17
	V = 93			^b,25^NA	0.97	–
				^a,6^T = 450	0.98	3
				V = 20		
				^c,27^T = N/A	0.97	20.8
				V = 24		
Lung Right	T = 177	0.98	3.04	^b,21^N/A	0.96	6.71
	V = 95			^b,25^NA	0.97	–
				^a,6^T = 450	0.98	3
				V = 20		
				^c,27^T = N/A	0.97	21.2
				V = 24		
Brachial Plexus Left	T = 58	0.53	6.88	^c,29^T = N/A	0.53	–
	V = 47			V = 1		
Brachial Plexus Right	T = 56	0.50	7.29	^c,28^T = N/A	0.31	18.97
	V = 43			V = 2		
				^b,28^T = N/A	0.26	20.06
				V = 2		
				^c,29^T = N/A	0.53	–
				V = 1		
Proximal Bronchial Tree	T = 88	0.82	4.23	–	–	–
	V = 100					
Spinal Cord	T = 105	0.90	1.62	^b,21^N/A	0.86	2.38
	V = 100			^b,25^NA	0.74	–
				^a,6^T = 450	0.82	4
				V = 20		
				^c,27^T = N/A	0.71	21.4
				V = 24		
Trachea	T = 143	0.91	2.27	^c,30^T = N/A	0.79	6
	V = 100			V = 10		
				^c,27^T = N/A	0.93	7.6
				V = 24		
GTV	T = 96	0.71	5.23	^a,8^T = 442	0.82	–
	V = 85			V = 544		
				^a,9^T = 681	0.68–0.74	2.60–7.94
				V = 2669		

a Deep learning-based auto-segmentation study.

b Human inter-observer variability study.

c Non-deep learning based auto-segmentation study.

Type of segmentation study, number of training cases (T), and number of validation (V) cases are listed where relevant. (GTV, gross tumor volume).

We observed that the DCs in our study approximated CCs (high DSC and low 95% HD) for the majority of the lung SABR OARs, with results comparable to other DC studies. For example, the heart DSC and 95% HD was 0.95 and 5.09mm using the current model trained with 191 contours and validated with 100 contours; this result is improved over a DSC and 95% HD of 0.90 and 13mm from a model that used 450 cases for training and 20 cases for validation (6), and approximates manual contouring IOV (0.93 and 6.42mm) (21). Similar comparisons to other studies are noted in **Table 3** for lung and spinal cord DCs, the latter of which was compared to spinal canal CCs in most cases in our study.

The esophagus had a lower mean DSC of 0.81 compared to other OARs (>0.90). However, our esophagus DC model still appears to be at least on par with other auto-segmentation (6, 27) and IOV studies (21, 25). The esophagus being difficult to delineate with certainty when collapsed likely contributes to contouring IOV; therefore, using a higher number of training case may not significantly improve validation results as there may always be a proportion of users that will interpret the esophagus differently than the DC model. The esophagus DC model may still assist contouring in clinical workflow, but likely would be better suited for auto-segmentation of every second or third slice and then manually edited and interpolated.

The PBT was another structure with a lower mean DSC value than most of the other OARs. We were unable to find any literature evaluating auto-segmentation or manual IOV for the PBT, but it is likely that some degree of IOV exists since adjustments to PBT contours are commonly suggested on peer review (31). The appearance of bronchial segments on CT scans varies among patients, so it would be important to expose the DC model to as much of this variation as possible during training in order to have reliable performance during clinical use. Training with additional cases could improve the performance of this DC model, although careful review and editing of this structure would be needed regardless since even expert manual contours commonly require adjustments (31).

BP DCs had the worst similarity to CCs among the structures evaluated in this study with mean DSC and 95% HD of 0.52 and 7.08mm, respectively. The BP is not always well visualized, so contouring guidelines based on anatomic landmarks have been developed (23). Despite these guidelines, contouring variations still exist due to personal contouring preferences and differing interpretations. One study noted a mean DSC and 95% HD of 0.26 and 20.06mm from comparing Radiation Oncology resident contours with expert contour when both groups were instructed to contour according to guidelines (28). DSC comparisons of the four expert RO contours in that study ranged from 0.23 to 0.52, which is similar to our results.

On review of the BP CCs, a wide range of contouring practices were observed; we noted that approximately 65% of validation cases contoured the BP similar to guidelines (23), while the remaining cases contoured only the visualized BP or contoured non-consecutive slices. Because of this variation, there is unlikely to be a single BP DC model that will be satisfactory for all users and it might be more appropriate to view this DC model as a starting point that a user can edit and interpolate according to their clinical practice. Nevertheless, considering that our model had relatively poorer DC-CC comparison metrics and used a lower number of training cases compared to other OARs, we feel that training this model with additional contours would be reasonable.

Finally, the GTV was the remaining structure that was seen to have fairly poor DC-CC comparisons with a mean DSC and 95% HD of 0.71 and 5.23mm. Lung tumors will vary in size, location, and shape, so adequately exposing the DC model to lesions with differing characteristics during training is again thought to be necessary for accurate performance. It is probable that the 96 training contours did not encompass the same range of scenarios seen in the 85 validation contours. For example, many of the 11 instances of false negatives in which the model did not generate a DC for had GTVs adjacent to the chest wall or hilum, likely representing an undertrained area.

On the other hand, other GTV DC models that were trained using a larger number of contours still appear to have suboptimal DC-CC comparison metrics. For example, Cao et al. used 442 training contours and 544 validation contours for developing their GTV DC model and reported a mean DSC of 0.83 (8), while Jiang et al. used 681 training and 2669 validation contours for multiple GTV DC models and reported DSC and 95% HD metrics of 0.68 and 2.60mm (9). Therefore, training specifically with cases containing tumor characteristics associated with poor DC model performance or using novel approaches to DC training algorithms (32) may be needed to see improved similarity to manual GTV contours.

Common limitations of auto-segmentation studies can be attributed to the validation dataset characteristics. Using a validation dataset containing too few cases, inaccurate contours, or containing data that is closely related to the training dataset is generally not recommended (10), as this may result in favorable validation comparison metrics but poor performance in clinical practice. Other institutions may have differing contouring practices and patient populations from the training institution, so validating with external cases is important to help inform on the generalizability of the DC models (10).

Our validation dataset contained a mean of 86 contours of each structure, which is larger than previous auto-segmentation studies (6, 12, 27), and these contours were peer-reviewed and approved for patient treatments, so we could also assume that they represented data of sufficiently high quality. Since cases from one center were included in the training dataset, we evaluated DC model performance on radiotherapy plans contoured by radiation oncologists from another center to investigate whether DC-CCs comparisons differed depending on the validation case center of origin. As previously mentioned, statistical analysis was not performed because statistically significant differences in contour comparisons would not necessarily imply that the DCs would not be clinically useful (4, 26).

Similar mean DC-CC comparison metrics were seen between center A and center B for OARs. The increased range of BP performance seen with center A compared to center B (**Figure 2**) was likely due to differences in the BP contouring practices observed between physicians at these two centers on our review of these cases. A larger difference was seen for the GTV, with the mean DSC being 0.76 for center A and 0.66 for center B. We did not match patient and tumor characteristics when selecting validation cases from each center, so we suspect that this difference is likely related to an unbalanced proportion of cases with specific tumor characteristics that were under-represented in the training dataset rather than a difference in image quality or contouring practices between the two centers.

A similar subset analysis was done to evaluate if DC accuracy differed on center A free-breathing and average 4D CT image sets. We did not observe any noticeable differences in the comparison metrics between these two groups.

While these subset analyses evaluate a smaller number of cases, their results support the external applicability of our DC models that included retrospective radiotherapy planning contours for training. However, few training and validation cases were scanned with IV contrast, so the applicability of our DC models on contrast CT scans is unknown and the presence of IV contrast could either improve or detract from model performance. Furthermore, while the validation contours were peer-reviewed and approved for patient treatment, they were not additionally reviewed or adjusted for purposes of this study. The presence of manual contouring errors that would not have impacted treatment planning but may have influenced the DC-CC comparison results could therefore not be excluded.

## Conclusions

With increasing interest in deep learning across Radiation Oncology (33, 34), auto-segmentation solutions are frequently being explored for use in radiotherapy planning (7). This study adds to the limited literature available intended to aid in the development of these algorithms. We found that the DCs in this study appear to perform comparably to other auto-segmentation algorithms, suggesting that existing radiotherapy planning contours, which are widely available at any institution, can likely be efficiently leveraged to create DC models for many OARs. These models will potentially have acceptable performance at multiple institutions, but further clinical workflow testing for confirmation is warranted and currently planned.

DCs for structures that tend to have more IOV or variation due to anatomic or disease factors were observed to perform less accurately. Such DC models may still benefit from using retrospective radiotherapy data during development, but ensuring a sufficiently large number of training cases, targeting additional training to areas of poor performance, and/or exploring other deep learning training approaches may be needed for adequate performance. Additional validation studies could further investigate the impact of specific training dataset characteristics, such as the number of training cases used or the proportion of retrospective data used.

Knowledge of being able to utilize existing high quality treatment plans in the development of auto-segmentation and our findings will hopefully facilitate the growth and uptake of machine learning auto-segmentation applications in Radiation Oncology clinical practices.

## Data Availability Statement

The datasets presented in this article are not readily available due to patient confidentiality. Requests to access the datasets should be directed to jordan.wong@bccancer.bc.ca.

## Ethics Statement

Written informed consent was not obtained from the individual(s) for the publication of any potentially identifiable images or data included in this article.

## Author Contributions

All authors were involved in the conception of this study and its design. JW, JAG, CK, and VH prepared the data used for developing the auto-segmentation models. JW, JAG, CK, VH, and TT prepared the data used to validate the auto-segmentation models. JW, JAG, CK, and JG performed the data analysis described in this study. JW was a major contributor in writing the manuscript. All authors contributed to the article and approved the submitted version.

## Conflict of Interest

The commercial auto-segmentation software used was provided without cost through a research agreement with Limbus AI Inc. No financial support was provided for this study. JAG, JG, and CK are directors of Limbus AI Inc.

The remaining authors declare that the research was conducted in the absence of any commercial or financial relationships that could be construed as a potential conflict of interest.

## References

[1] Abreu CE Ferreira PP de Moraes FY Neves WF Jr Gadia R Carvalho Hde A. Stereotactic Body Radiotherapy in Lung Cancer: An Update. J Bras Pneumol (2015) 41(4):376–87. 10.1590/S1806-37132015000000034 PMC4635958 26398758

[2] Palma DA Olson R Harrow S Gaede S Louie AV Haasbeek C. Stereotactic Ablative Radiotherapy Versus Standard of Care Palliative Treatment in Patients With Oligometastatic Cancers (SABR-COMET): A Randomised, Phase 2, Open-Label Trial. Lancet (2019) 393(10185):2051–8. 10.1016/S0140-6736(18)32487-5 30982687

[3] Wong J Huang V Wells DM Giambattista JA Giambattista J Kolbeck C. Implementation of Deep Learning-Based Auto-Segmentation for Radiotherapy Planning Structures: A Multi-Center Workflow Study. Int J Radiat Oncol (2020) 108(3):S101. 10.1016/j.ijrobp.2020.07.2278 PMC8186196 34103062

[4] Cha E Elguindi S Onochie I Gorovets D Deasy JO Zelefsky M. Clinical Implementation of Deep Learning Contour Autosegmentation for Prostate Radiotherapy. Radiother Oncol (2021) 159:1–7. 10.1016/j.radonc.2021.02.040 33667591 PMC9444280

[5] Wong J Fong A McVicar N Smith S Giambattista J Wells D. Comparing Deep Learning-Based Auto-Segmentation of Organs at Risk and Clinical Target Volumes to Expert Inter-Observer Variability in Radiotherapy Planning. Radiother Oncol (2020) 144:152–8. 10.1016/j.radonc.2019.10.019 31812930

[6] Lustberg T van Soest J Gooding M Peressutti D Aljabar P van der Stoep J. Clinical Evaluation of Atlas and Deep Learning Based Automatic Contouring for Lung Cancer. Radiother Oncol (2018) 126(2):312–7. 10.1016/j.radonc.2017.11.012 29208513

[7] Brouwer CL Dinkla AM Vandewinckele L Crijns W Claessens M Verellen D. Machine Learning Applications in Radiation Oncology: Current Use and Needs to Support Clinical Implementation. Phys Imaging Radiat Oncol (2020) 16:144–8. 10.1016/j.phro.2020.11.002 PMC7807598 33458358

[8] Cao H Liu H Song E Hung CC Ma G Xu X. Dual-Branch Residual Network for Lung Nodule Segmentation. Appl Soft Comput (2020) 86:105934. 10.1016/j.asoc.2019.105934

[9] Jiang J Hu YC Liu CJ alpenny D Hellmann MD Deasy JO. Multiple Resolution Residually Connected Feature Streams for Automatic Lung Tumor Segmentation From CT Images. IEEE Trans Med Imag (2019) 38(1):134–44. 10.1109/TMI.2018.2857800 PMC6402577 30040632

[10] Vandewinckele L Claessens M Dinkla A Brouwer C Crijns W Verellen D. Overview of Artificial Intelligence-Based Applications in Radiotherapy: Recommendations for Implementation and Quality Assurance. Radiother Oncol (2020) 153:55–66. 10.1016/j.radonc.2020.09.008 32920005

[11] El Naqa I Ruan D Valdes G Dekker A McNutt T Ge Y. Machine Learning and Modeling: Data, Validation, Communication Challenges. Med Phys (2018) vol. 45(10):e834–40. 10.1002/mp.12811 PMC6181755 30144098

[12] Noothout J de Vos BD Wolterink J Išgum I. Automatic Segmentation of Thoracic Aorta Segments in Low-Dose Chest CT. SPIE Med Imag (2018) 10574:105741S. 10.1117/12.2293114

[13] Wang J Lu J Qin G Shen L Sun Y Ying H. Technical Note: A Deep Learning-Based Autosegmentation of Rectal Tumors in MR Images. Med Phys (2018) 45(6):2560–4. 10.1002/mp.12918 29663417

[14] Ronneberger O Fischer P Brox T. Convolutional Networks for Biomedical Image Segmentation. International Conference on Medical image computing and computer-assisted intervention (2015) 234–41. 10.1007/978-3-319-24574-4_28.

[15] Nikolov S Blackwell S Mendes R De Fauw J Meyer C Hughes C. Deep Learning to Achieve Clinically Applicable Segmentation of Head and Neck Anatomy for Radiotherapy. arXiv preprint (2018). Available at: http://arxiv.org/abs/1809.04430 (Accessed November 8, 2018).

[16] Abadi M Agarwal A Barham P Brevdo E Chen Z Citro C. Tensorflow: Large-Scale Machine Learning on Heterogeneous Distributed Systems. arXiv preprint (2016). Available at: http://arxiv.org/abs/1603.04467 (Accessed November 15, 2018).

[17] Clark K Vendt B Smith K Freymann J Kirby J Koppel P. The Cancer Imaging Archive (TCIA): Maintaining and Operating a Public Information Repository. J Digit Imag (2013) 26(6):1045–57. 10.1007/s10278-013-9622-7 PMC3824915 23884657

[18] Yang J Sharp G Veeraraghavan H van Elmpt W Dekker A Lustberg T. Data From Lung Ct Segmentation Challenge. Cancer Imaging Arch (2017). 10.7937/K9/TCIA.2017.3r3fvz08

[19] National Cancer Institute Clinical Proteomic Tumor Analysis Consortium. Radiology Data From the Clinical Proteomic Tumor Analysis Consortium Lung Squamous Cell Carcinoma [Cptac-Lscc] Collection [Data Set]. Cancer Imaging Arch (2018). 10.7937/k9/tcia.2018.6emub5l2

[20] National Cancer Institute Clinical Proteomic Tumor Analysis Consortium. Radiology Data From the Clinical Proteomic Tumor Analysis Consortium Lung Adenocarcinoma [Cptac-LUAD] Collection [Data Set]. Cancer Imaging Arch (2018). 10.7937/k9/tcia.2018.pat12tbs

[21] Yang J Veeraraghavan H Armato SG 3rd Farahani K Kirby JS Kalpathy-Kramer J. Autosegmentation for Thoracic Radiation Treatment Planning: A Grand Challenge at AAPM 2017. Med Phys (2018) 45(10):4568–81. 10.1002/mp.13141 PMC6714977 30144101

[22] Hugo GD Weiss E Sleeman WC Balik S Keal PJ Lu J. Data From 4D Lung Imaging of NSCLC Patients. Cancer Imaging Arch (2016). 10.7937/K9/TCIA.2016.ELN8YGLE

[23] Hall WH Guiou M Lee NY Dublin A Narayan S Vijayakumar S. Development and Validation of a Standardized Method for Contouring the Brachial Plexus: Preliminary Dosimetric Analysis Among Patients Treated With IMRT for Head-and-Neck Cancer. Int J Radiat Oncol Biol Phys (2008) 72(5):1362–7. 10.1016/j.ijrobp.2008.03.004 18448267

[24] Kong F-M Quint L Machtay M. Atlases for Organs at Risk (Oars) in Thoracic Radiation Therapy. Radiation Therapy Oncology Group (2019). Available at: https://www.rtog.org/LinkClick.aspx?fileticket=qlz0qMZXfQs%3D&tabid=361 (Accessed October 26, 2019).

[25] Tsang Y Hoskin P Spezi E Landau D Lester J Miles E. Assessment of Contour Variability in Target Volumes and Organs at Risk in Lung Cancer Radiotherapy. Tech Innov Patient Support Radiat Oncol (2019) 10:8–12. 10.1016/j.tipsro.2019.05.001 32095541 PMC7033767

[26] Zabel WJ Conway JL Gladwish A Skliarenko J Didiodato G Goorts-Matthews L. Clinical Evaluation of Deep Learning and Atlas-Based Auto-Contouring of Bladder and Rectum for Prostate Radiation Therapy. Pract Radiat Oncol (2021) 11(1):e80–9. 10.1016/j.prro.2020.05.013 32599279

[27] Wittenstein O Hiepe P Sowa LH Karsten E Fandrich I Dunst J. Automatic Image Segmentation Based on Synthetic Tissue Model for Delineating Organs at Risk in Spinal Metastasis Treatment Planning. Strahlenther Und Onkol (2019) 195(12):1094–103. 10.1007/s00066-019-01463-4 PMC6868111 31037351

[28] Awan M Dyer BA Kalpathy-Cramer J Bongers E Dahele M Yang J. Auto-Segmentation of the Brachial Plexus Assessed With TaCTICS-A Software Platform for Rapid Multiple-Metric Quantitative Evaluation of Contours. Acta Oncol (Madr) (2015) 54(4):557–60. 10.3109/0284186X.2014.953638 PMC4801518 25279958

[29] Van de Velde J Wouters J Vercauteren T De Gersem W Achten E De Neve W. Optimal Number of Atlases and Label Fusion for Automatic Multi-Atlas-Based Brachial Plexus Contouring in Radiotherapy Treatment Planning. Radiat Oncol (2016) 11(1). 10.1186/s13014-015-0579-1 PMC4705618 26743131

[30] Ayyalusamy A Vellaiyan S Subramanian S Ilamurugu A Satpathy S Nauman M. Auto-Segmentation of Head and Neck Organs at Risk in Radiotherapy and Its Dependence on Anatomic Similarity. Radiat Oncol J (2019) 37(2):134–42. 10.3857/roj.2019.00038 PMC6610007 31266293

[31] Lo AC Liu M Chan E Lund C Truong P Loewen S. The Impact of Peer Review of Volume Delineation in Stereotactic Body Radiation Therapy Planning for Primary Lung Cancer: A Multicenter Quality Assurance Study. J Thorac Oncol (2014) 9(4):527–33. 10.1097/JTO.0000000000000119 24736076

[32] Fong A Swift CL Wong J McVicar N Giambattista JA Kolbeck C. Automatic Deep Learning-Based Segmentation of Brain Metastasis on MPRAGE Mr Images for Stereotactic Radiotherapy Planning. Int J Radiat Oncol (2019) 105(1):E134. 10.1016/j.ijrobp.2019.06.2169

[33] Boldrini L Bibault J-E Masciocchi C Shen Y Bittner M-I. Deep Learning: A Review for the Radiation Oncologist. Front Oncol (2019) 9:977. 10.3389/fonc.2019.00977 31632910 PMC6779810

[34] Osman A. “Radiation Oncology in the Era of Big Data and Machine Learning for Precision Medicine”. In: Machine Learning in Medicine and Biology. London, United Kingdom: IntechOpen (2019). 10.5772/intechopen.84629

