# Supplementary material for: Training and Validation of Deep Learning-Based Auto-Segmentation Models for Lung Stereotactic Ablative Radiotherapy Using Retrospective Radiotherapy Planning Contours
Source: Front Oncol. 2021 Jun 7;11:626499. doi: 10.3389/fonc.2021.626499 (PMC8215371; doi:10.3389/fonc.2021.626499)
Supplement: Supplementary file 1 [file DataSheet_1.pdf]

Example Case 1

Auto-segmentation = Blue

Manual clinical contour = Purple

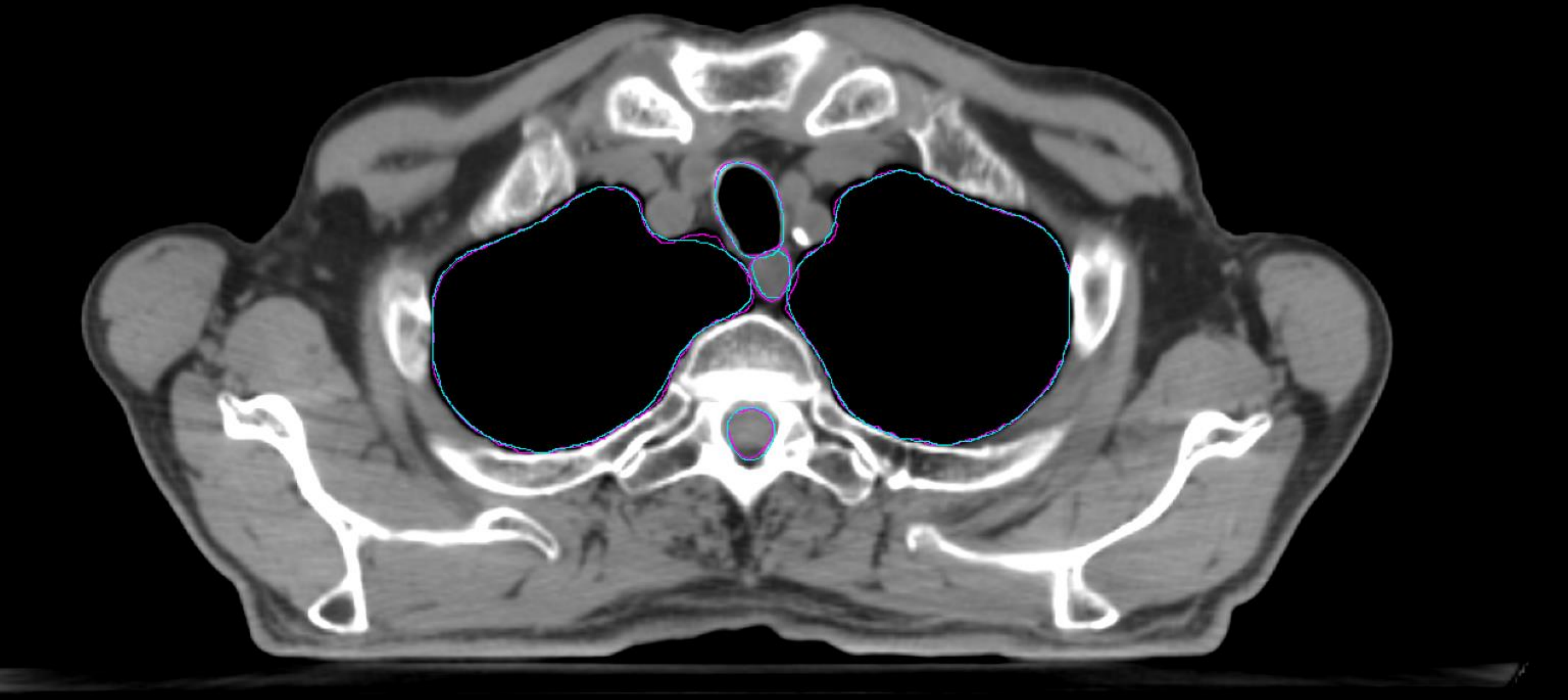

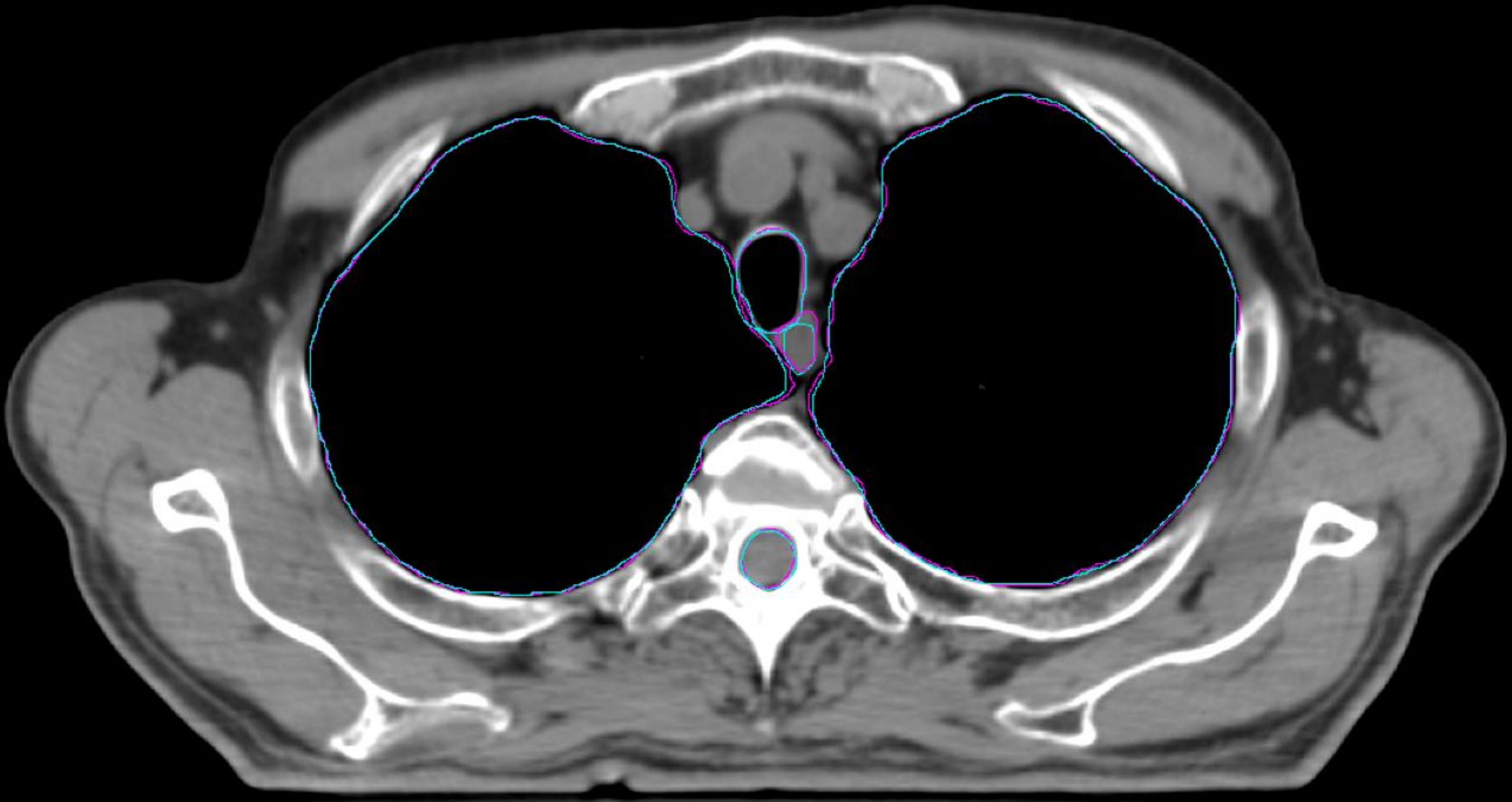

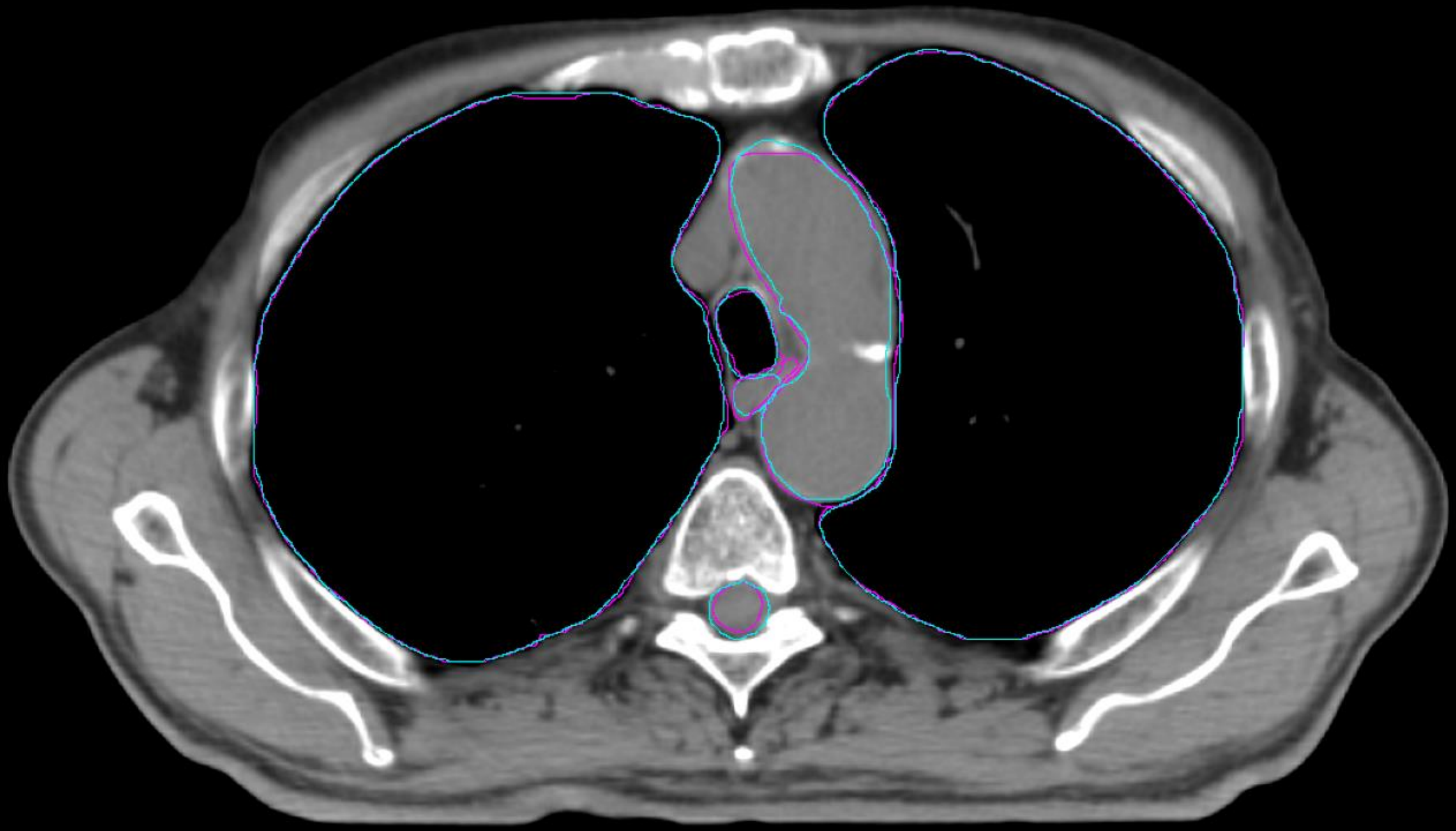

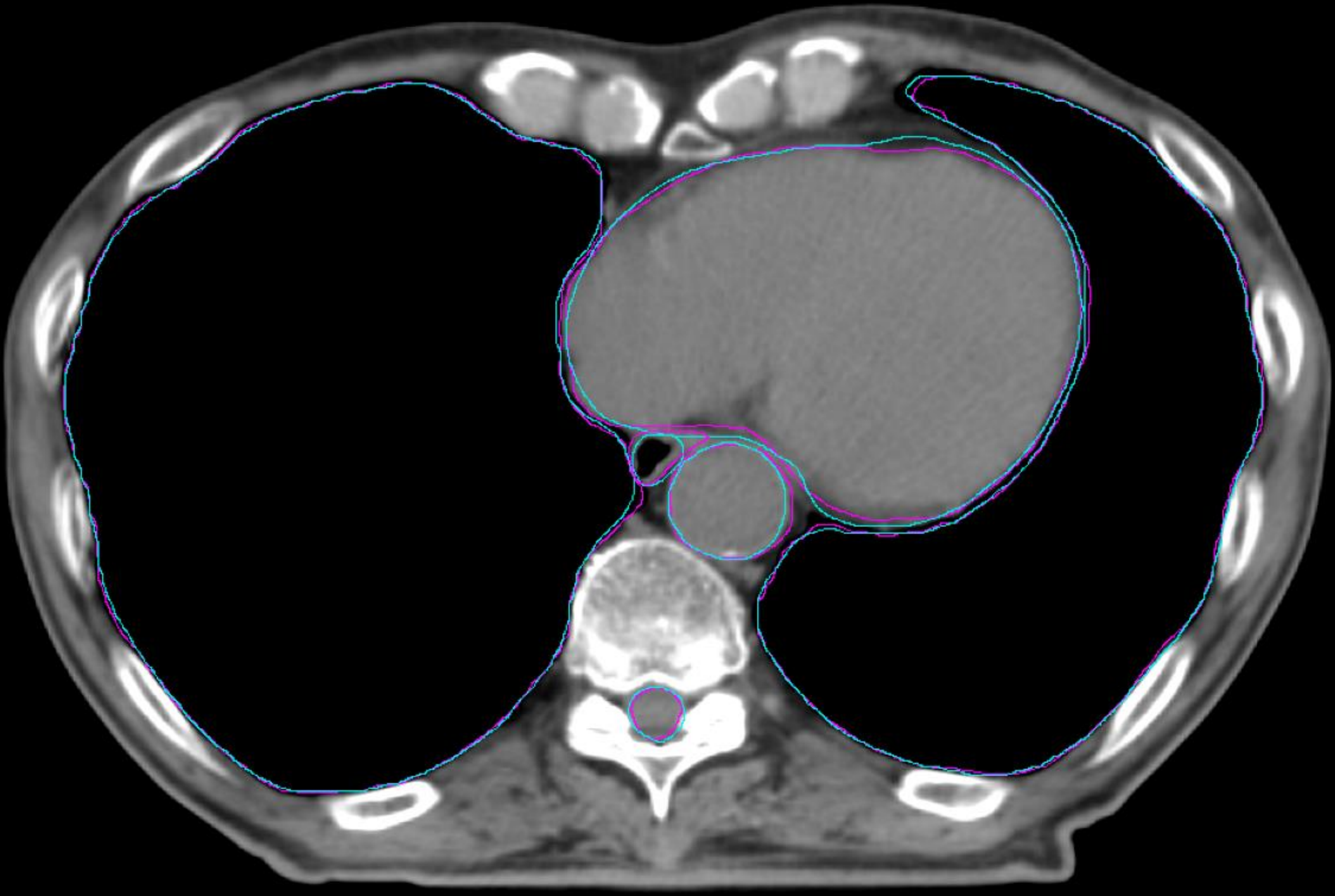

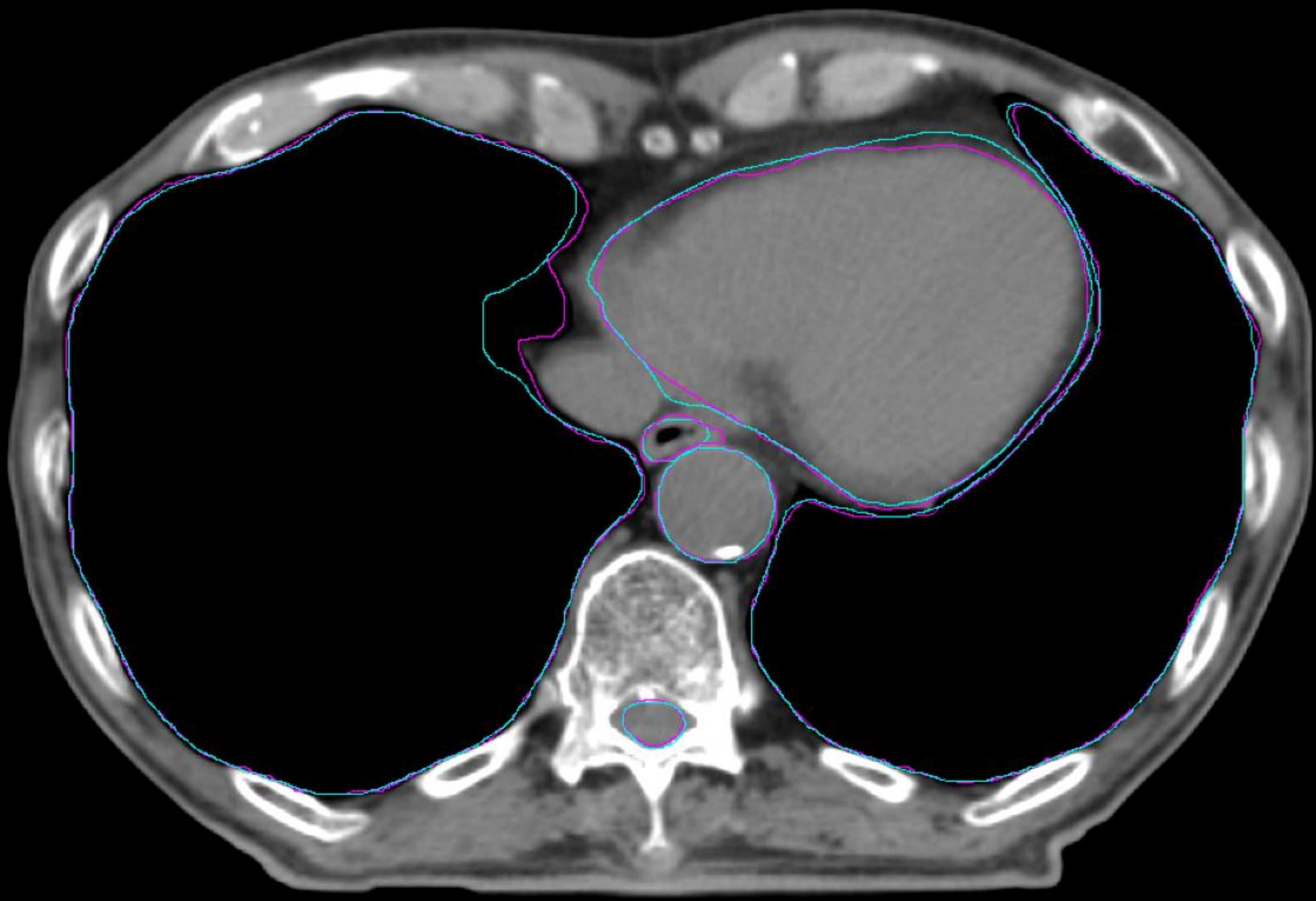

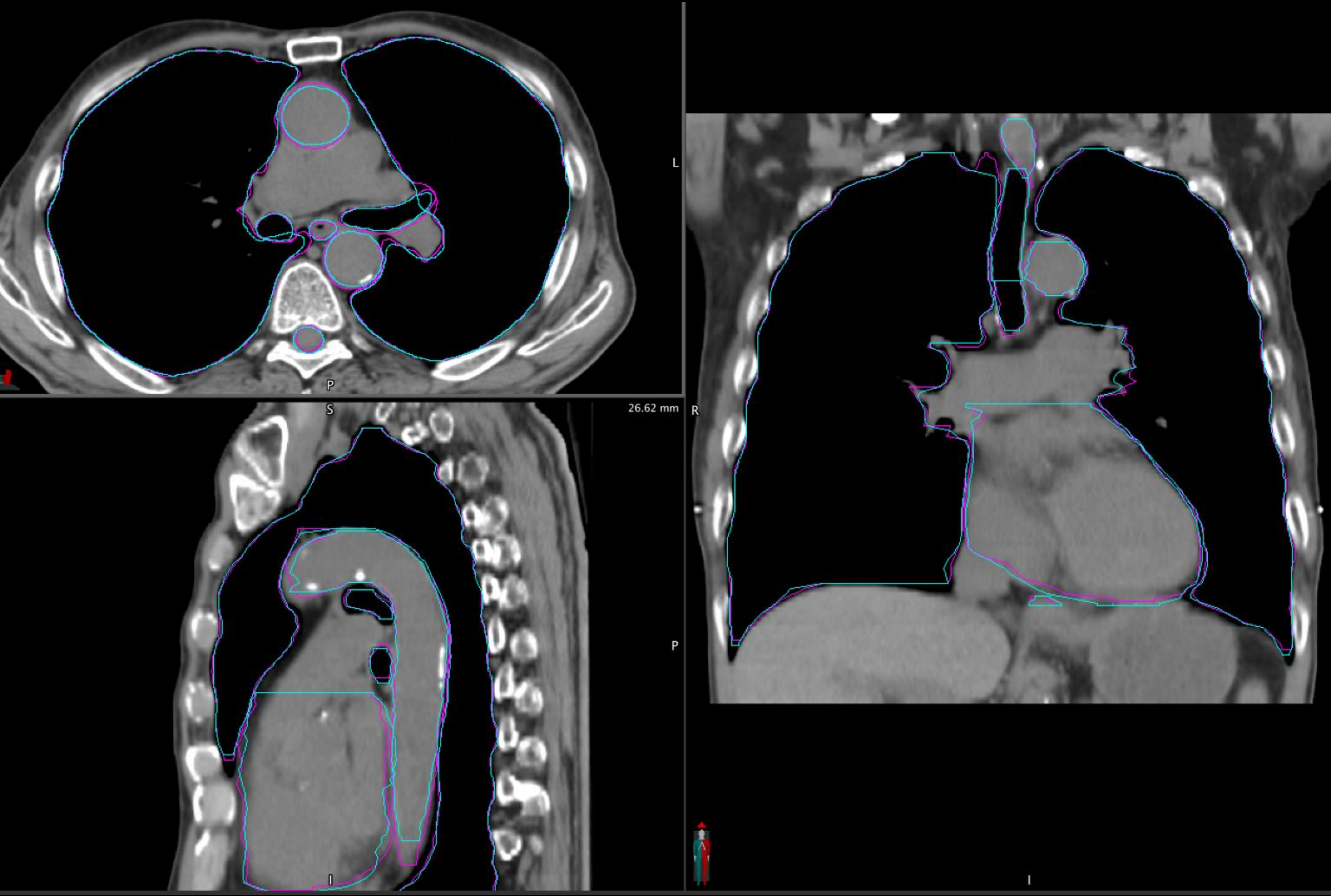

L
P
S
26.62 mm
R
P
I
I

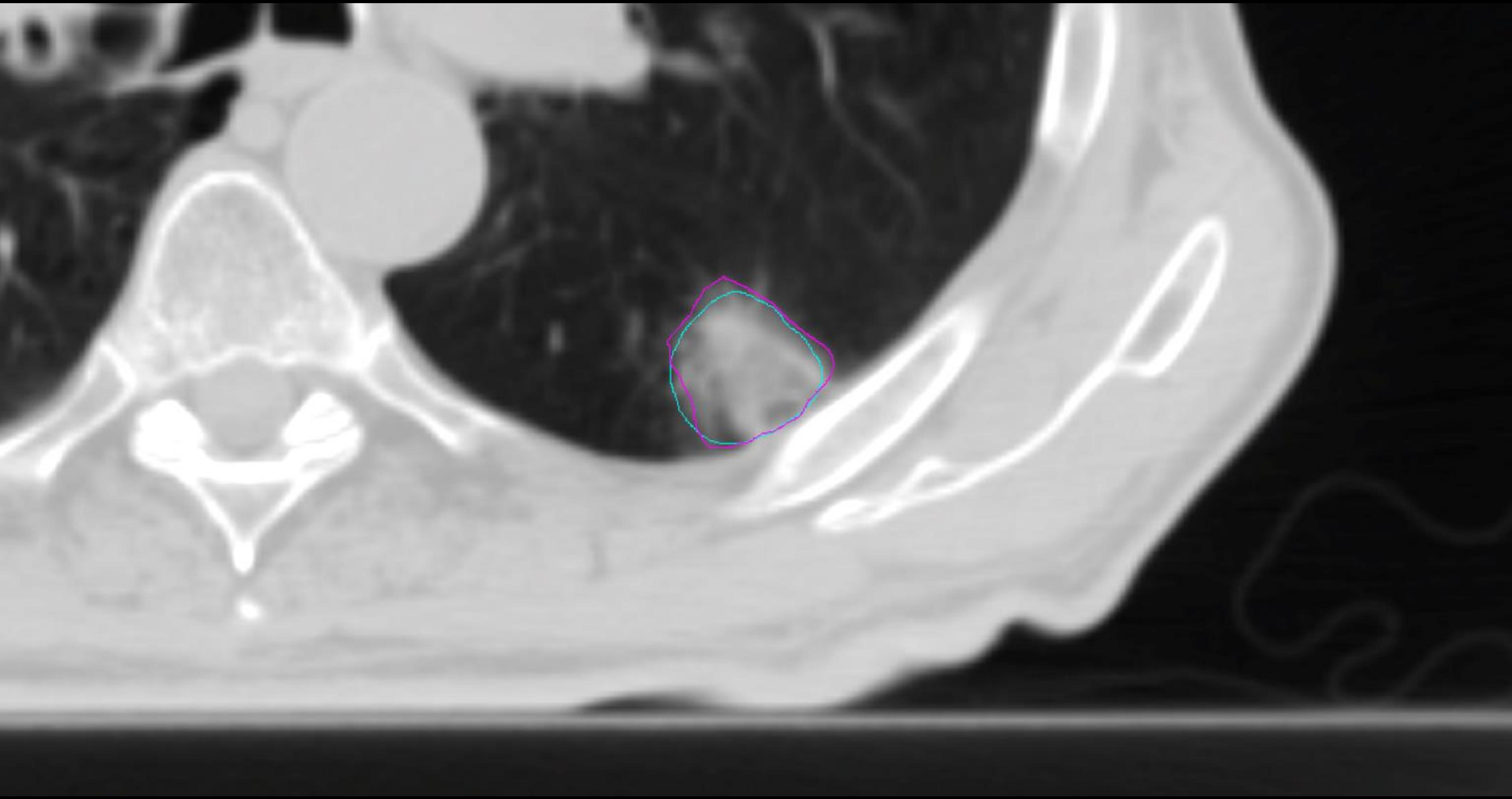

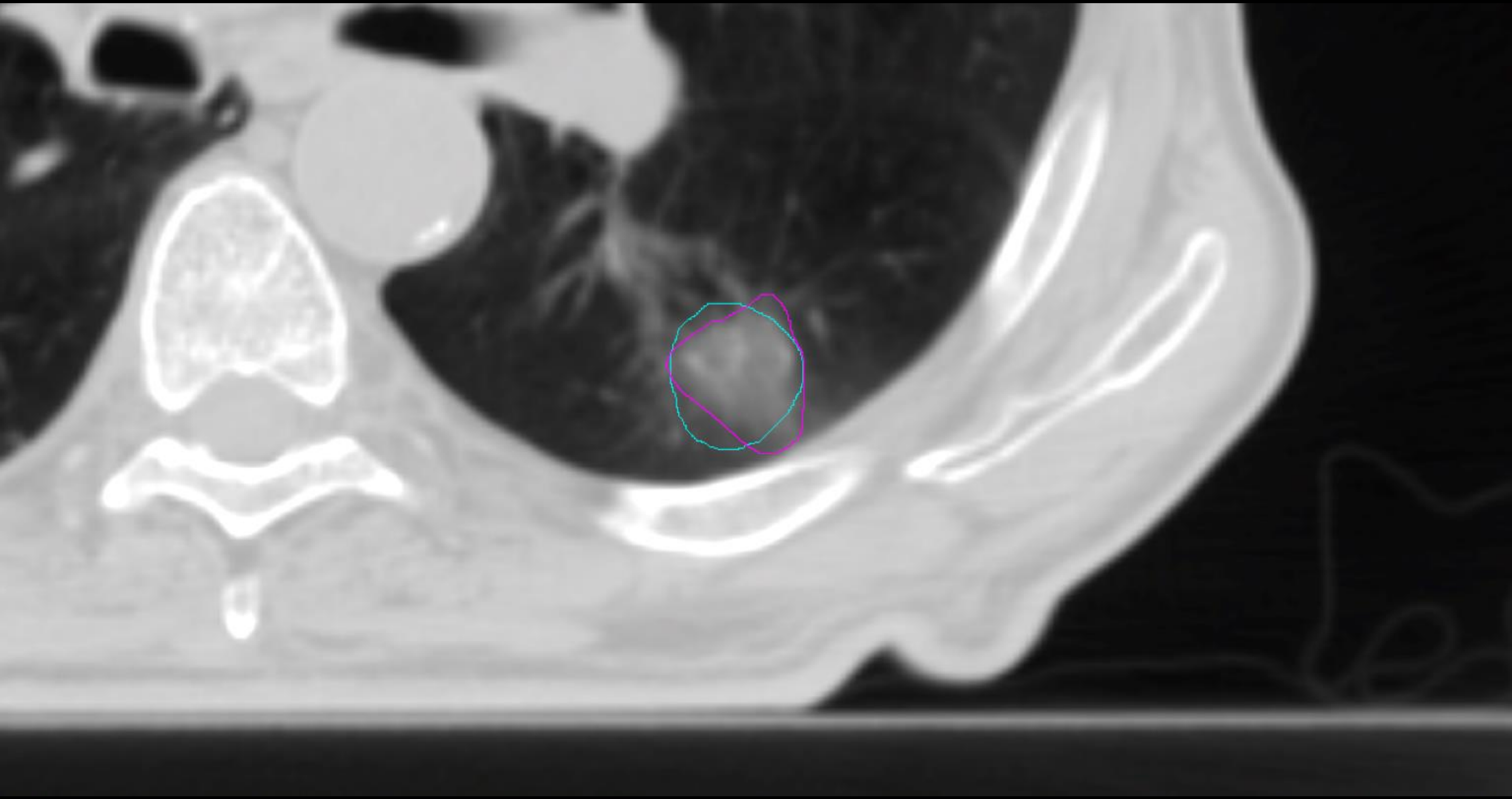

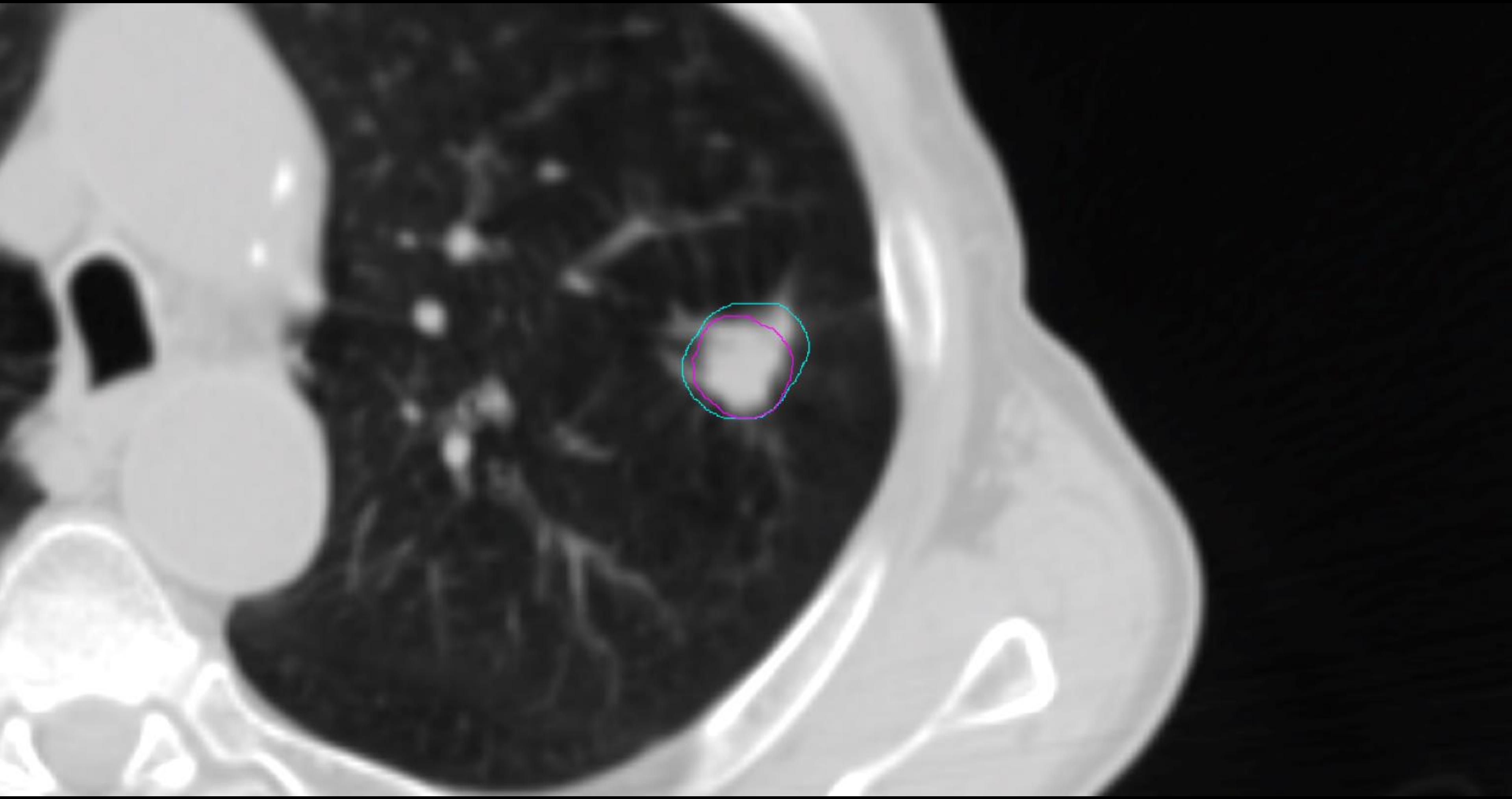

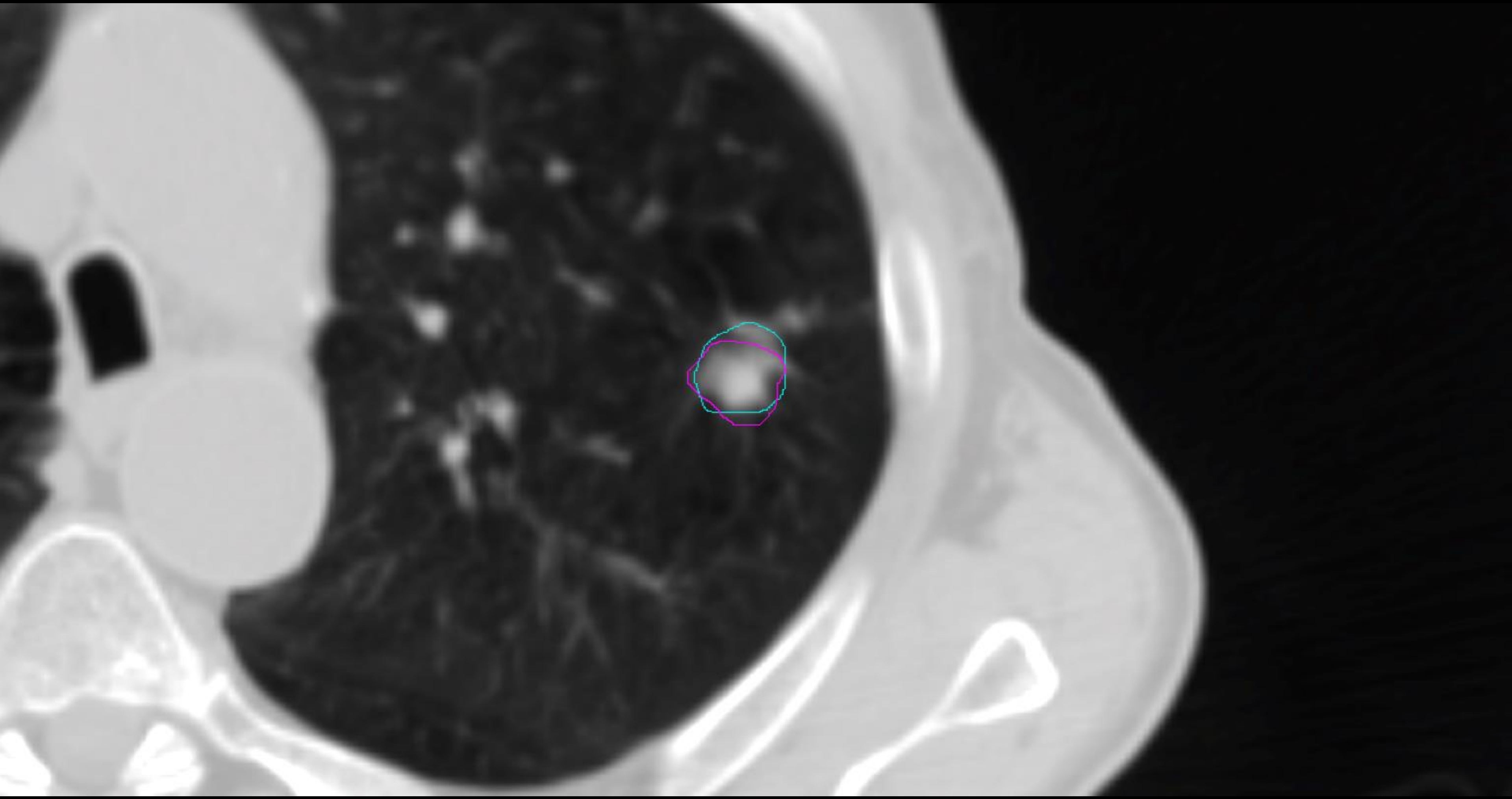

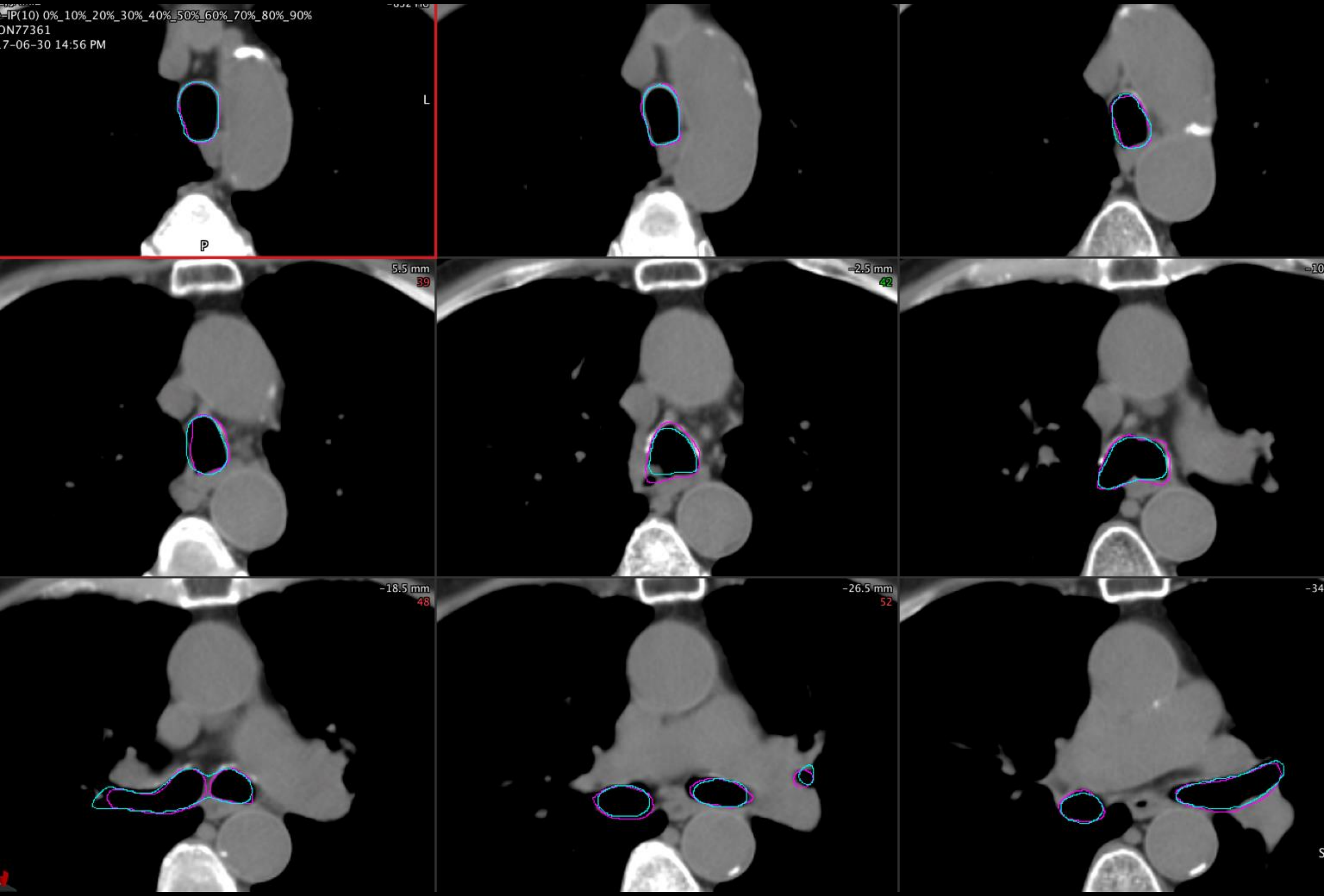

-IP(10) 0%_10%_20%_30%_40%_50%_60%_70%_80%_90%
ON77361
17-06-30 14:56 PM
L
P
5.5 mm
39
-2.5 mm
42
-18.5 mm
48
-26.5 mm
52

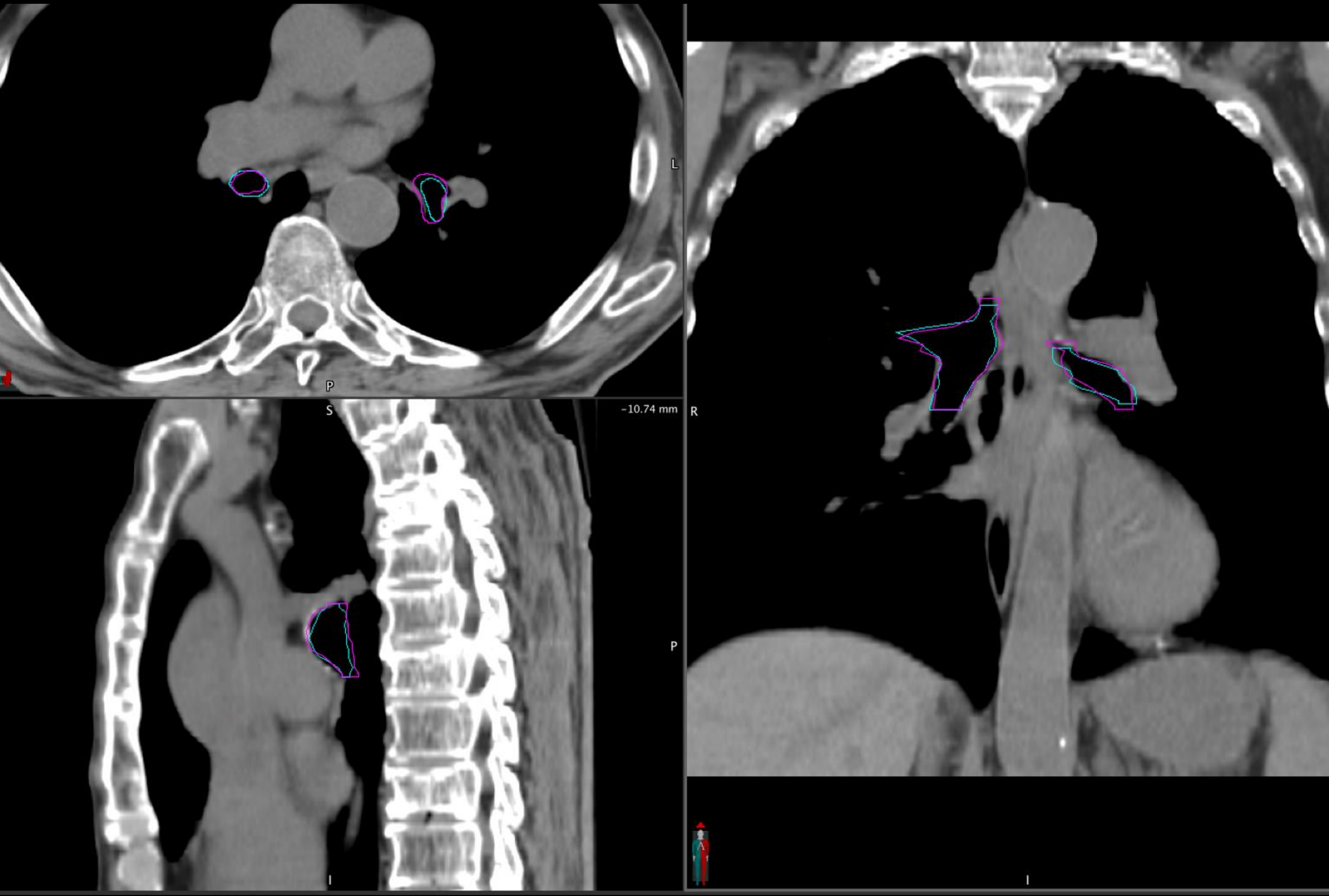

L
P
S
-10.74 mm
R
P
I
I

Example Case 2

Auto-segmentation = Blue

Manual clinical contour = Purple

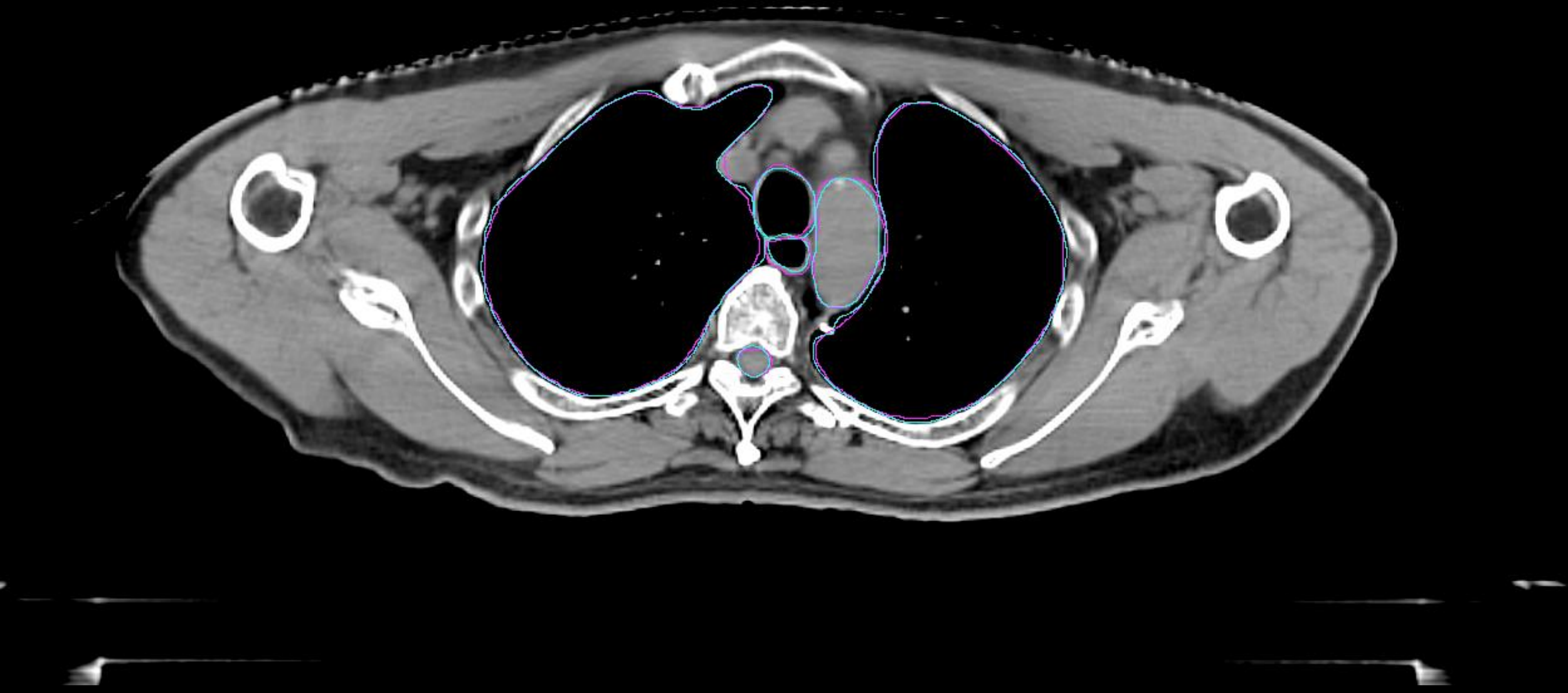

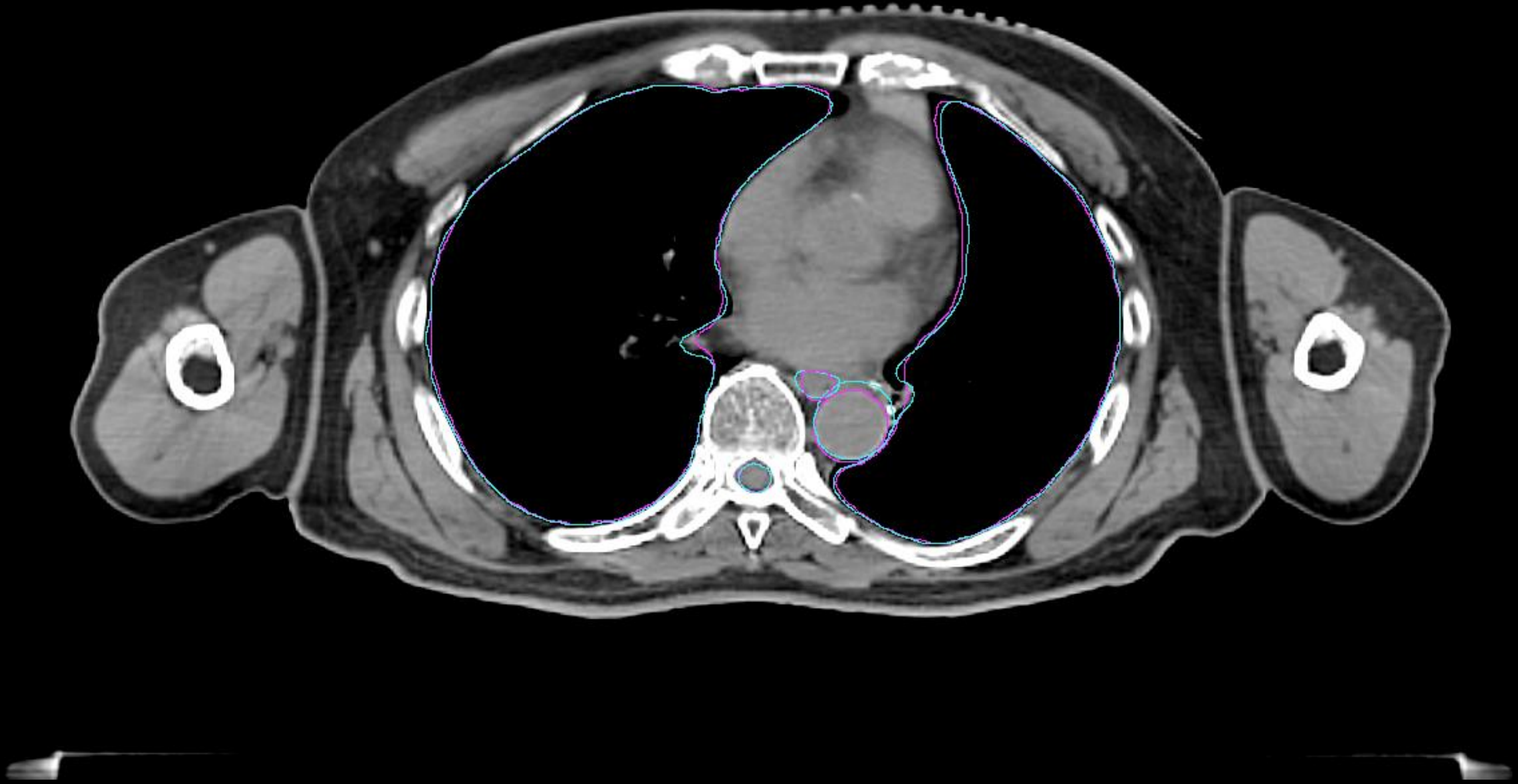

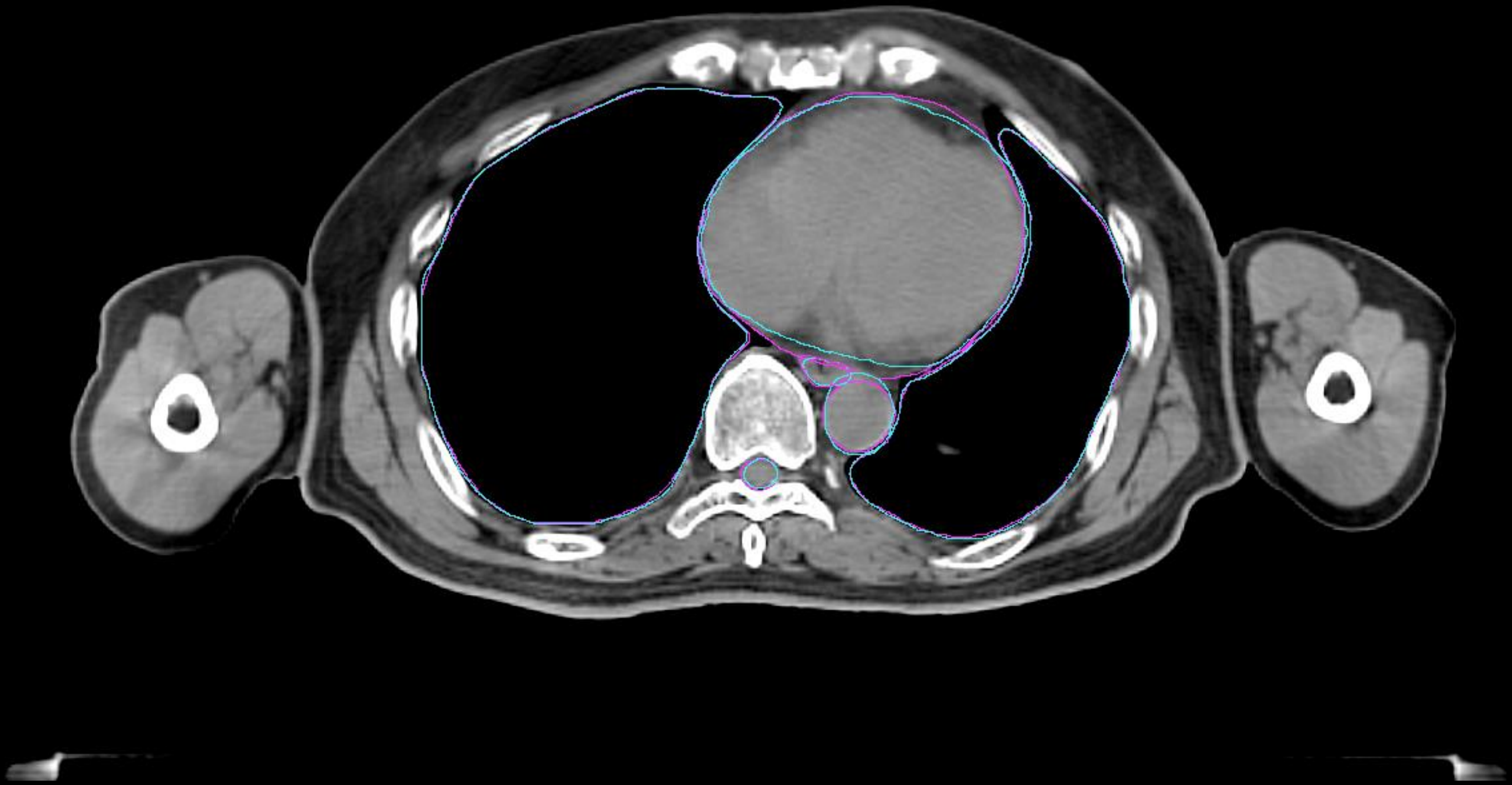

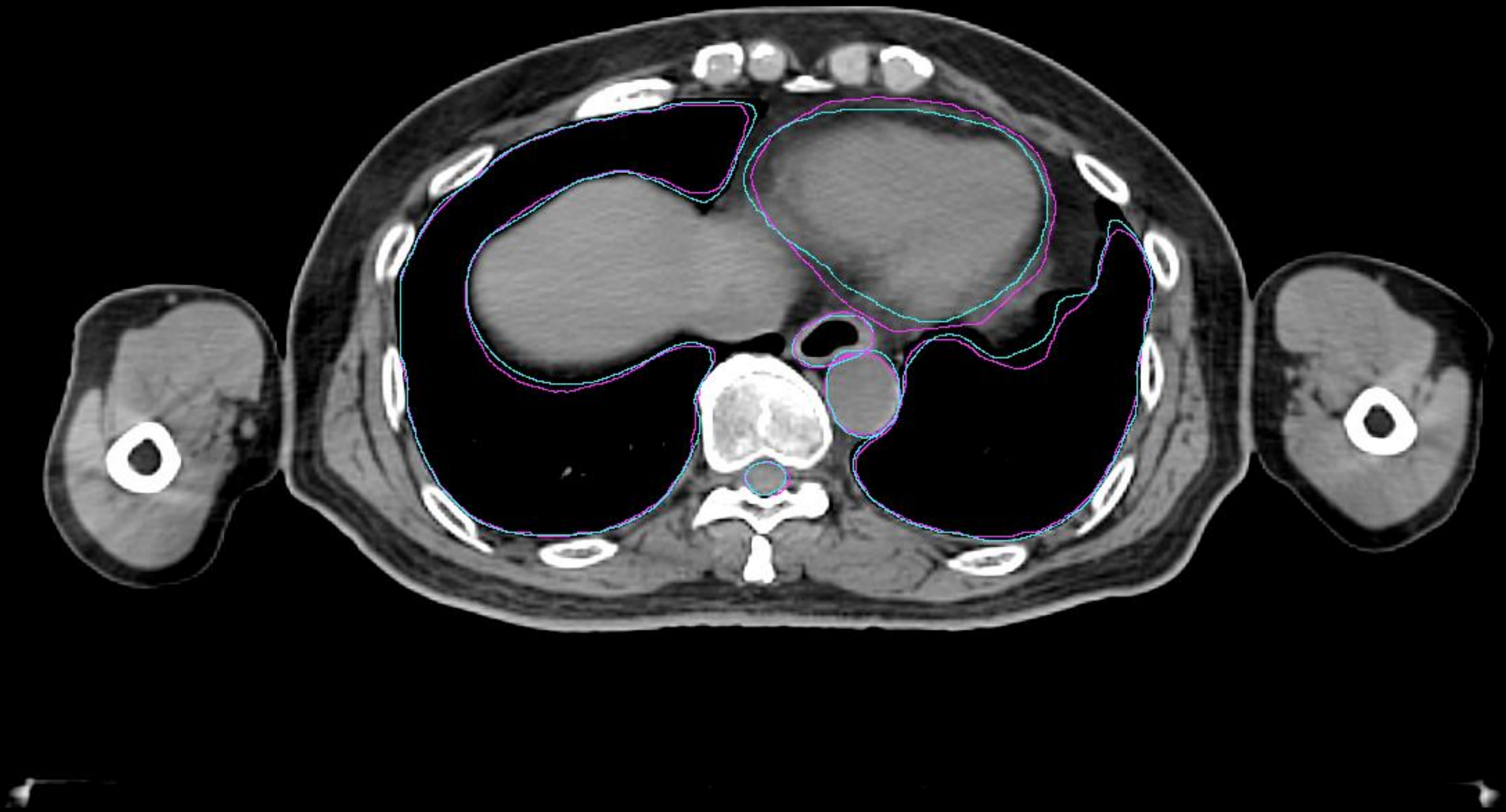

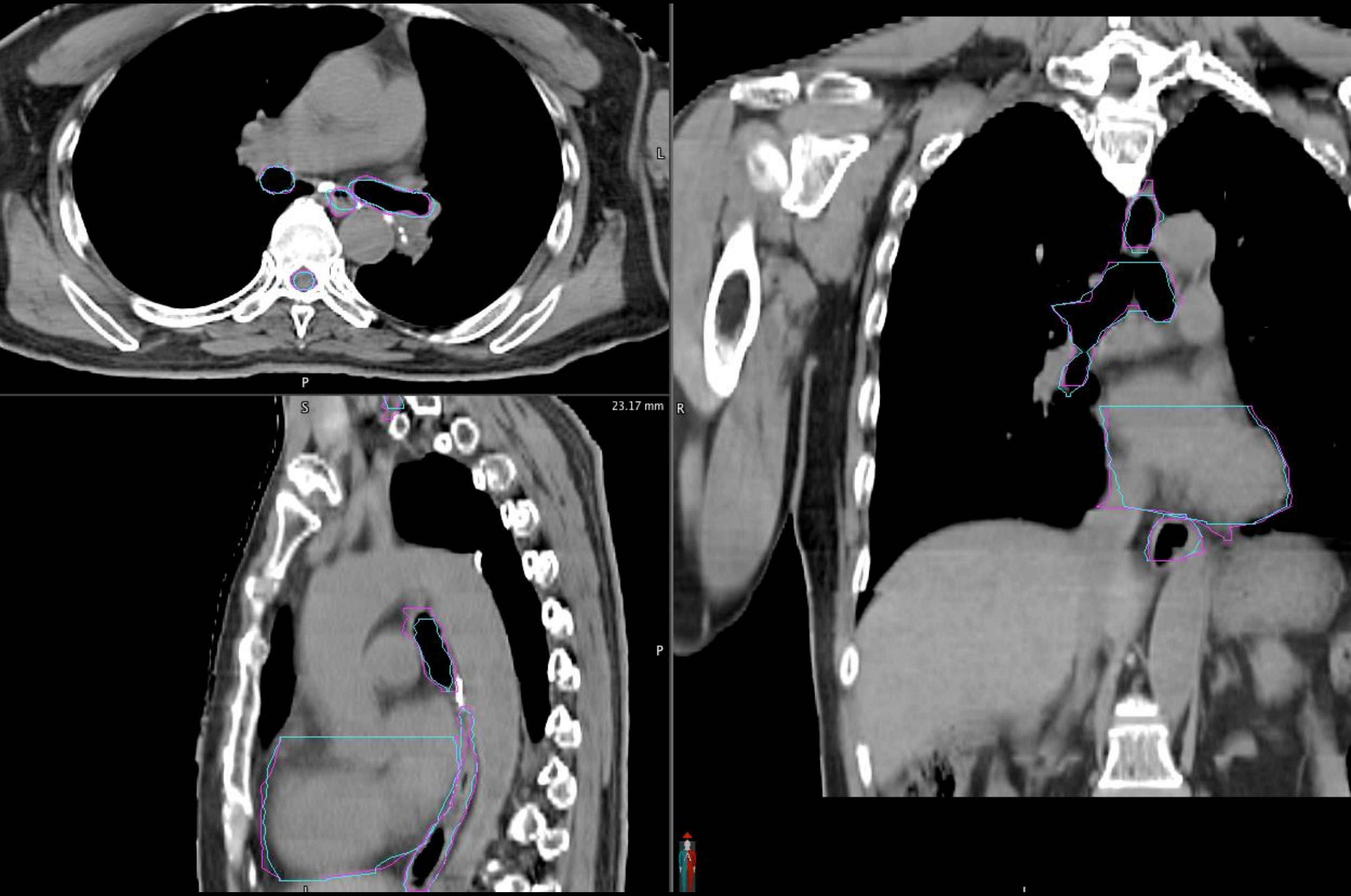
L
P
S
23.17 mm
P
R

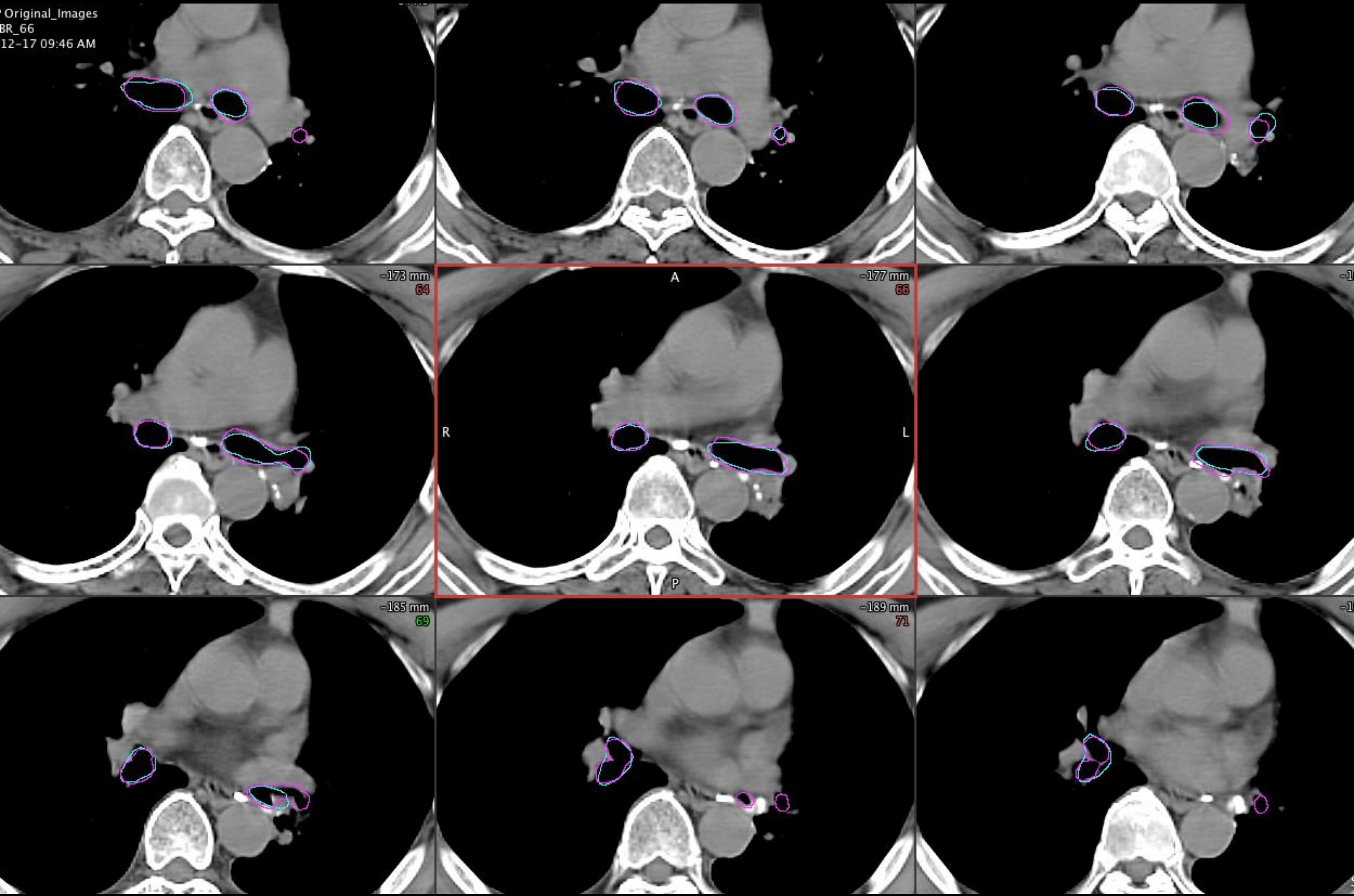
Original_Images
BR_66
12-17 09:46 AM
-173 mm
64
A
-177 mm
66
R
L
P
-185 mm
69
-189 mm
71

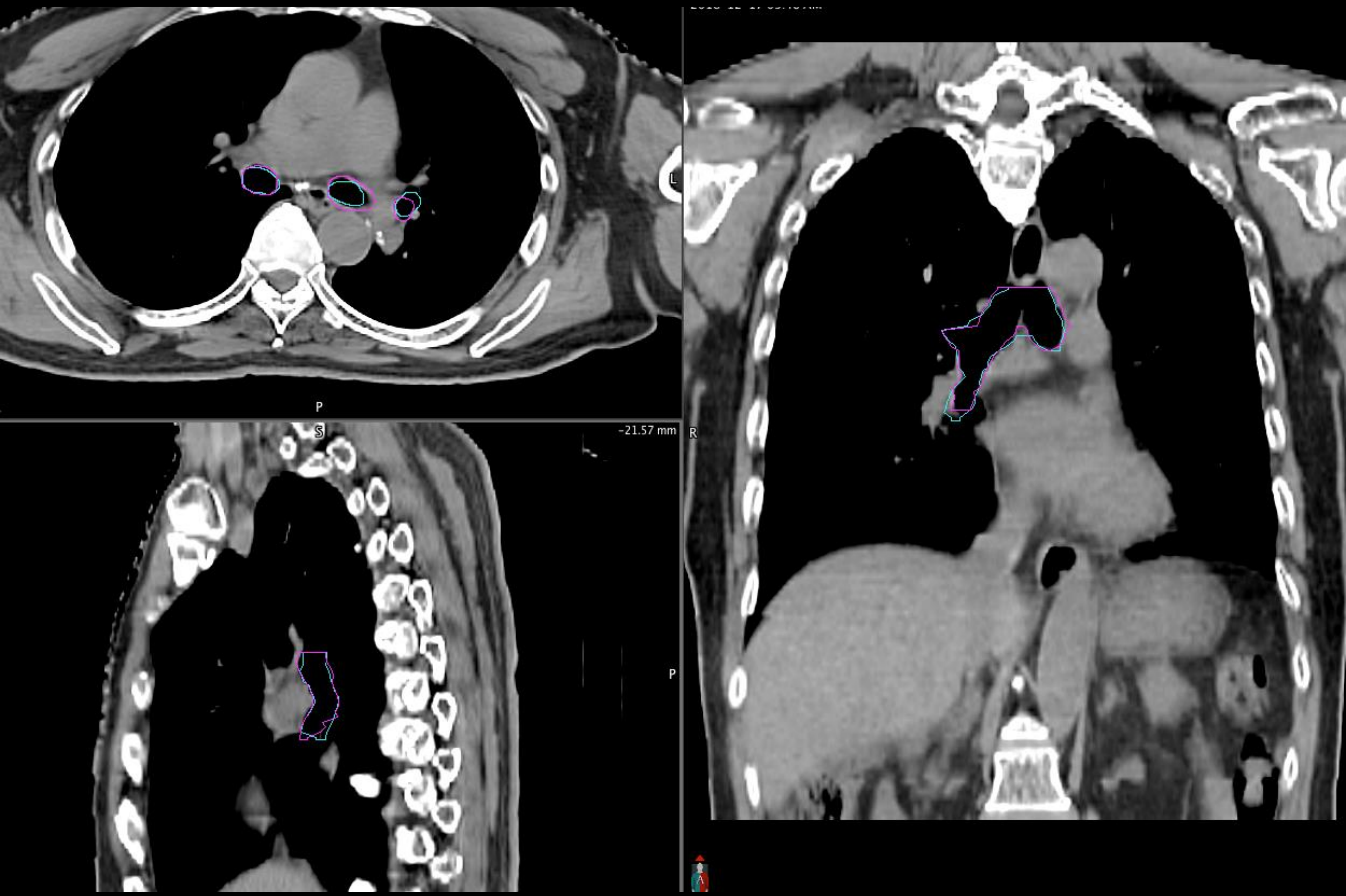
L
P
S
-21.57 mm
P
R

Additional Examples of Structures

Auto-segmentation = Blue

Manual clinical contour = Purple

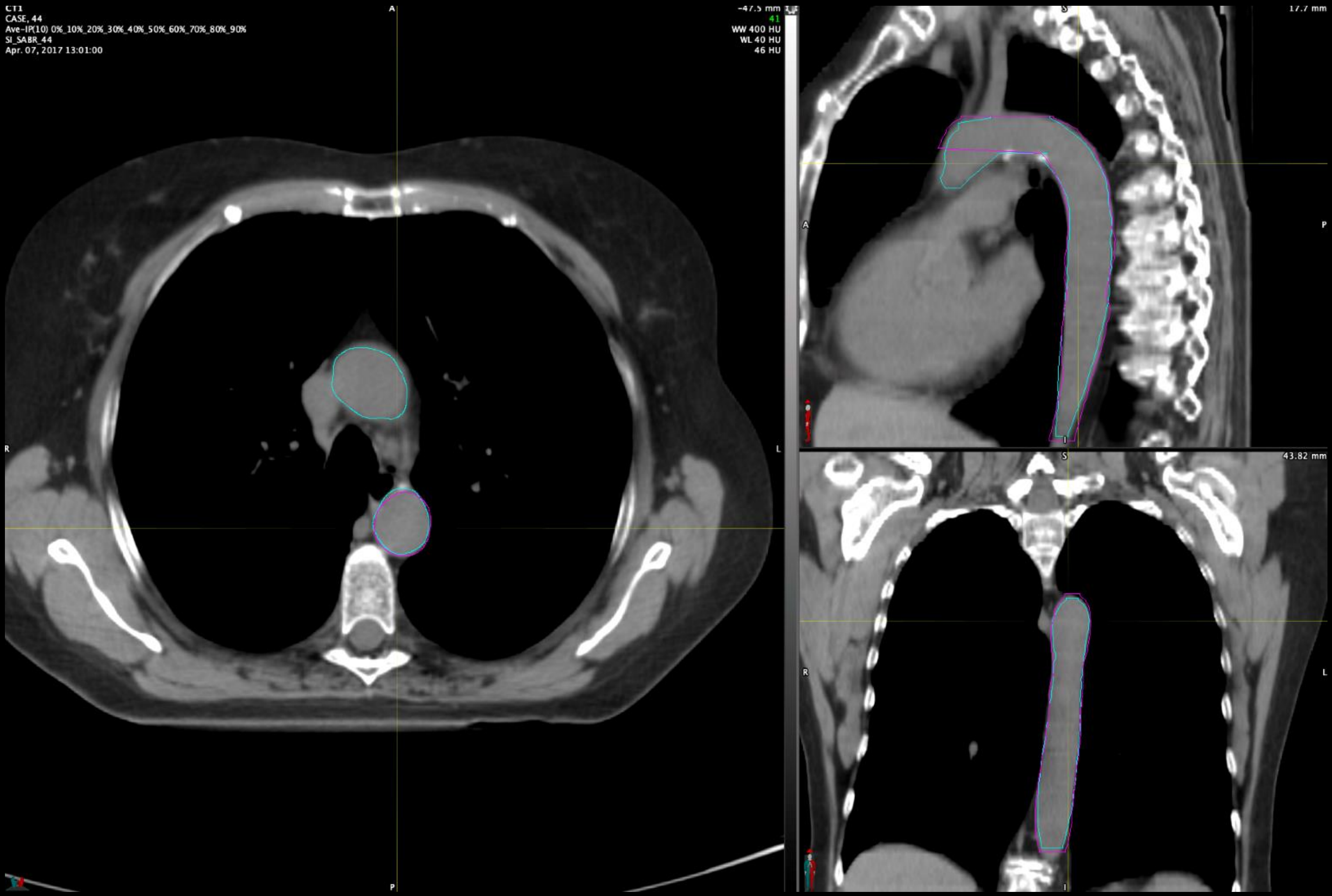

Aorta – 95% HD 3.95mm, DSC 0.85 (sup and inf borders cropped to same plane before comparison)

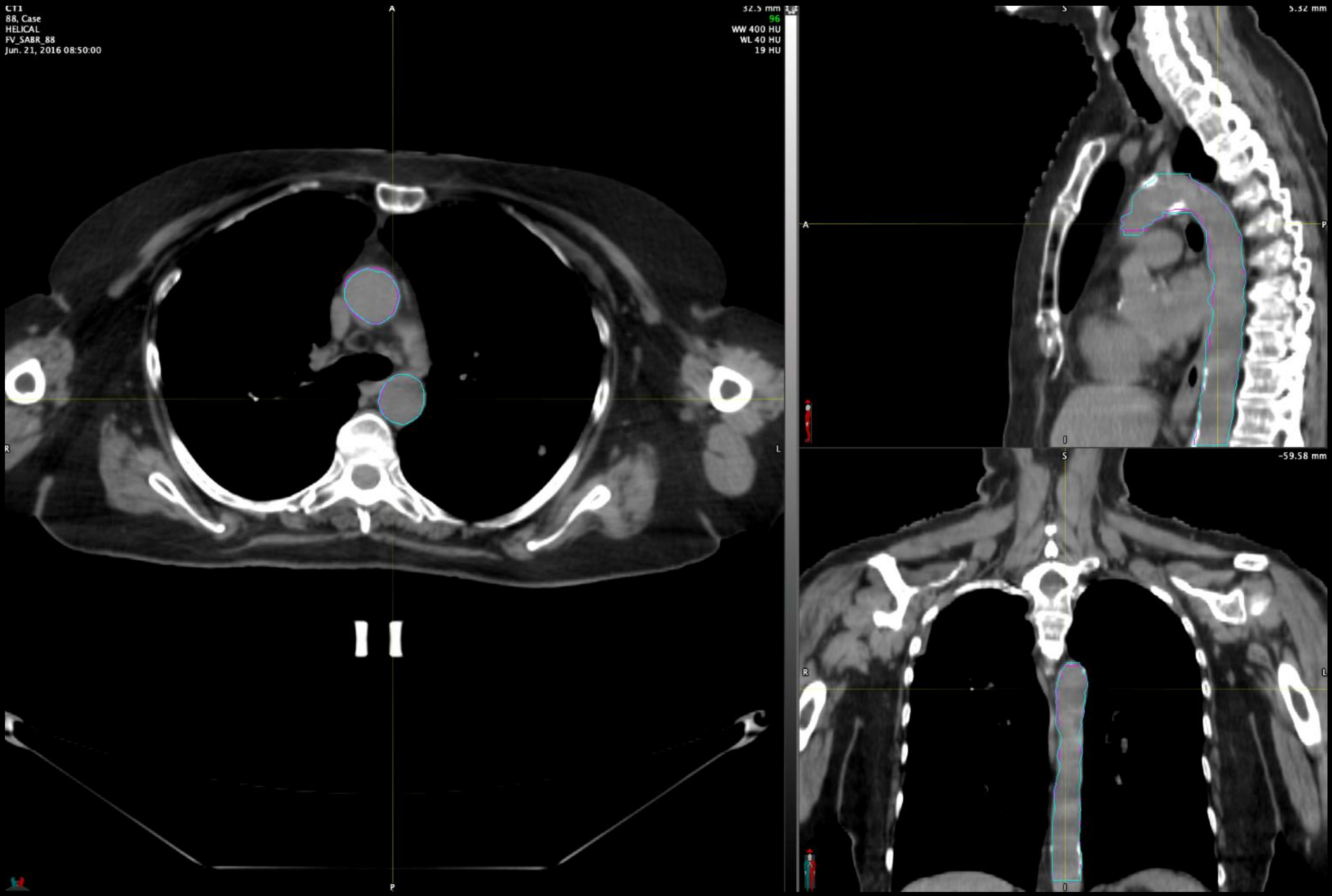

Aorta – 95% HD 1.64mm, DSC 0.98 (sup and inf borders cropped to same plane before comparison)

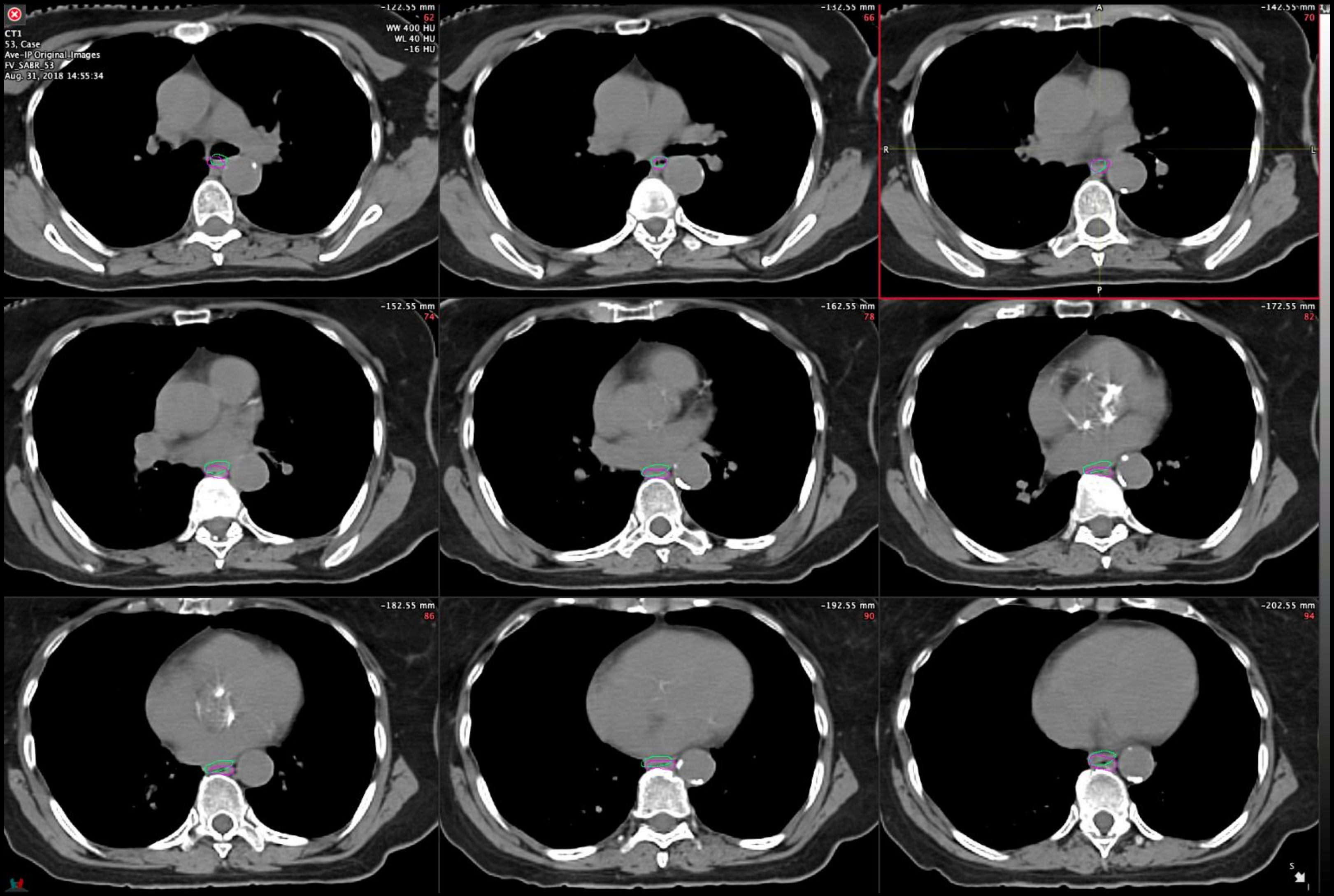

Esophagus – 95% HD 3.73mm, DSC 0.64

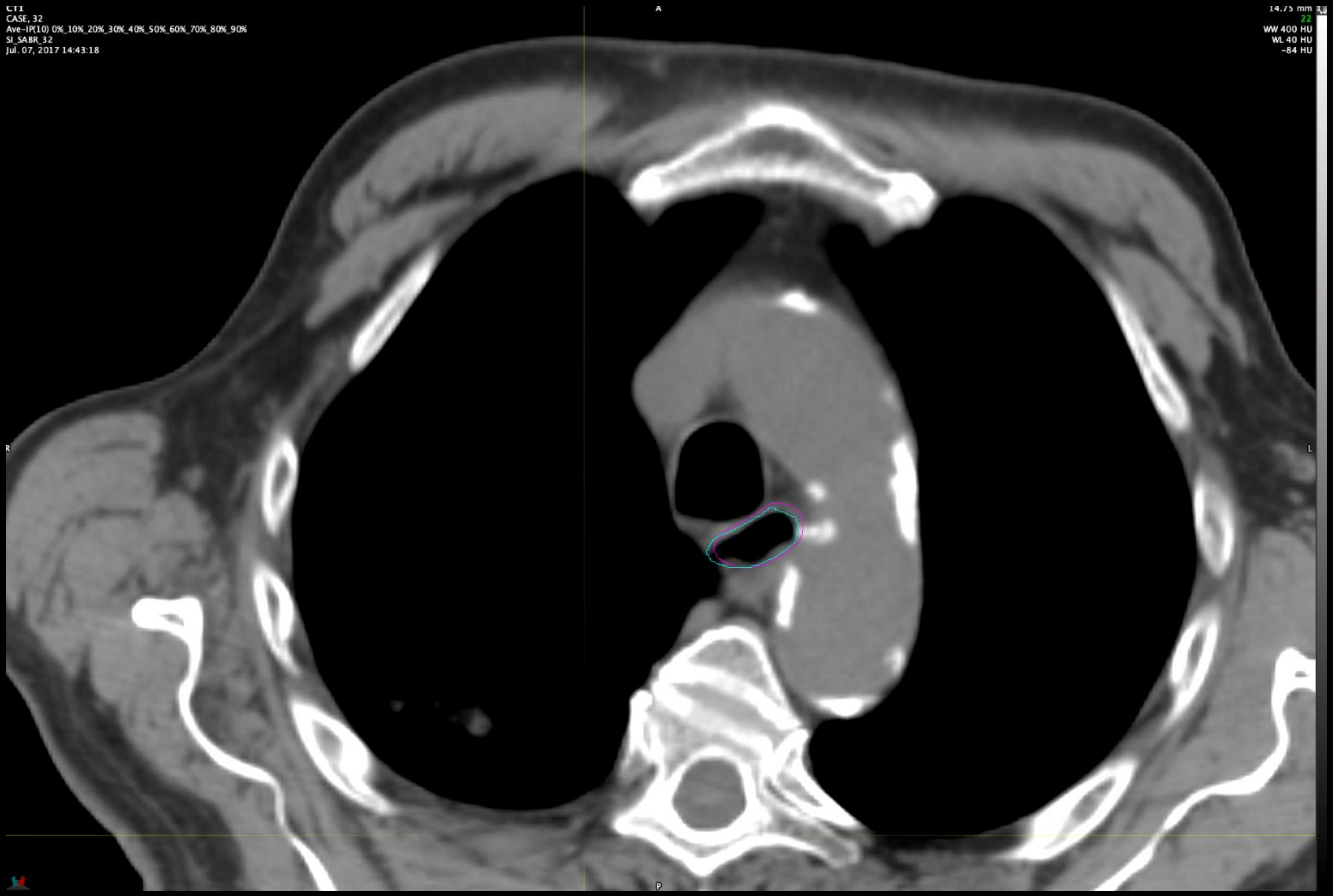

Esophagus – 95% HD 2.14mm, DSC 0.96

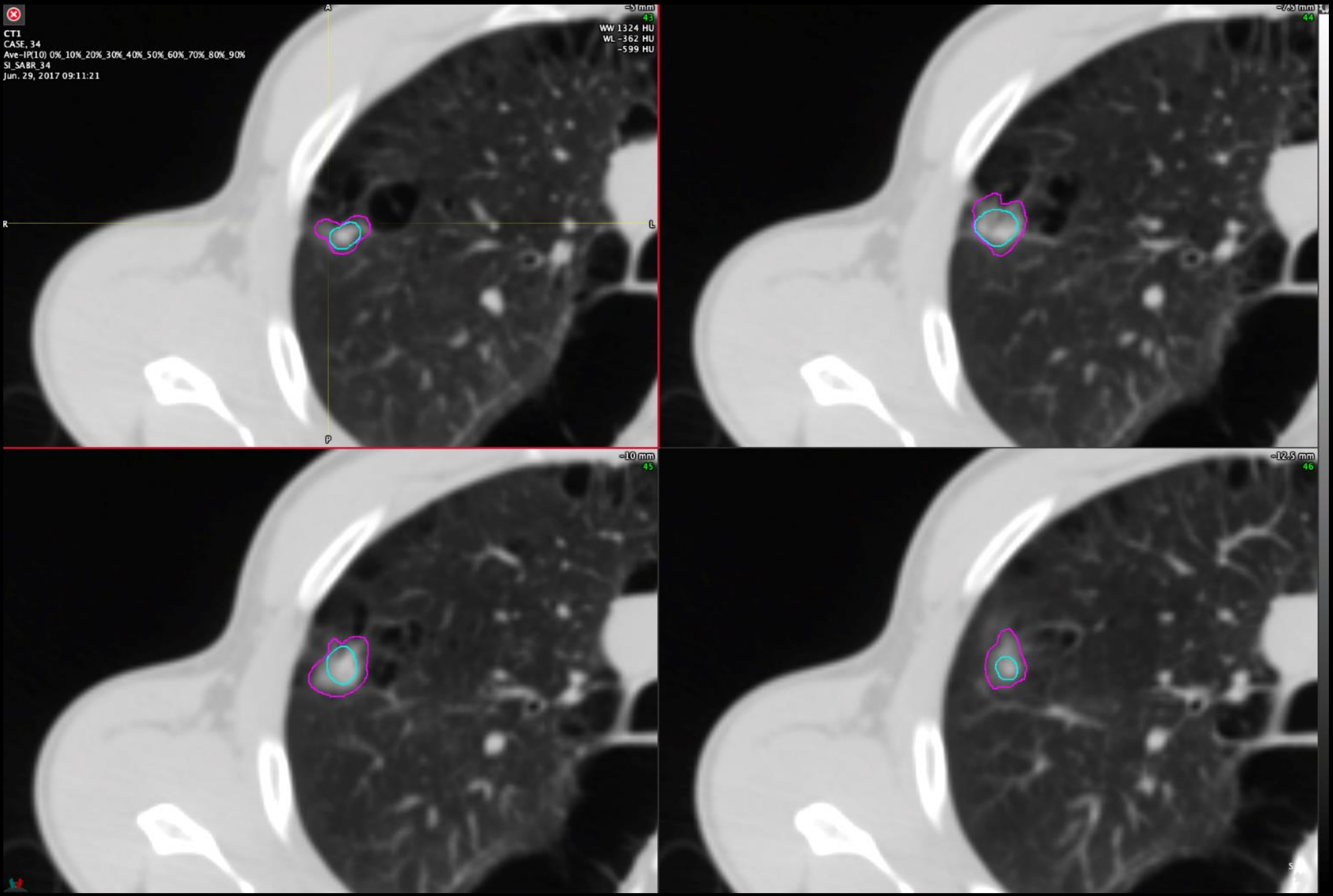

GTV – 95% HD 5.4mm, DSC 0.19

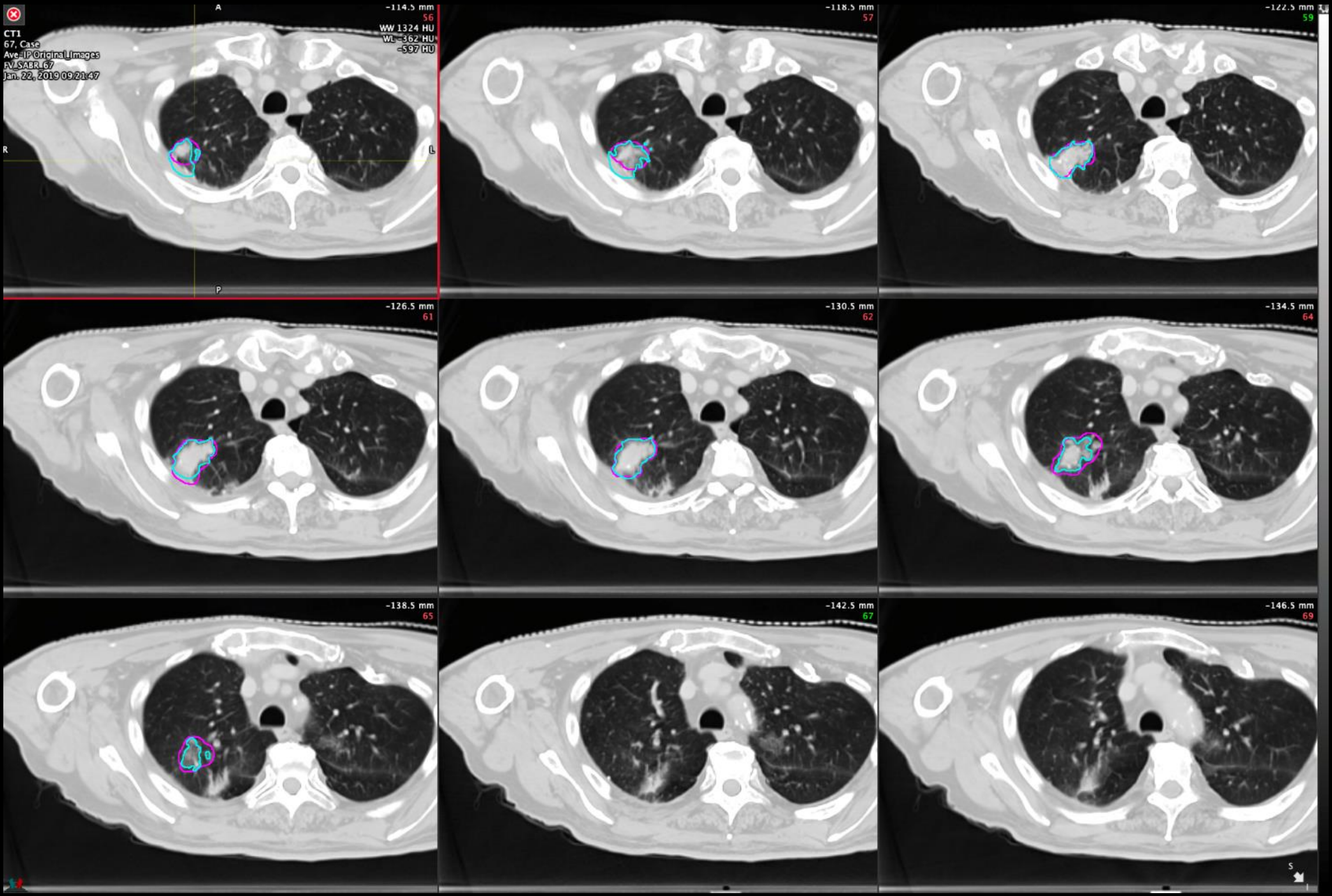

GTV – 95% HD 3.25mm, DSC 0.90

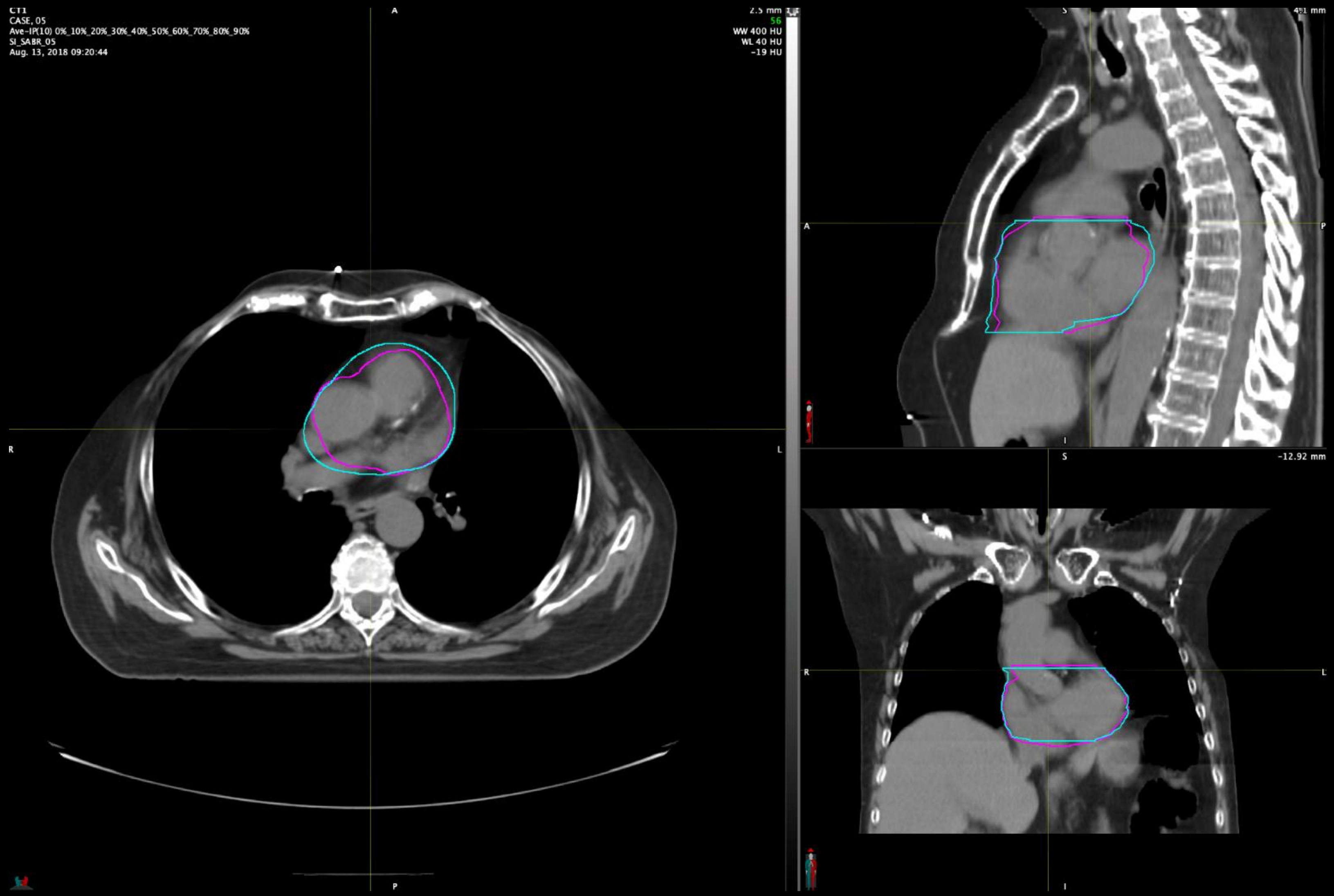

Heart – 95% HD 7.8mm, DSC 0.90

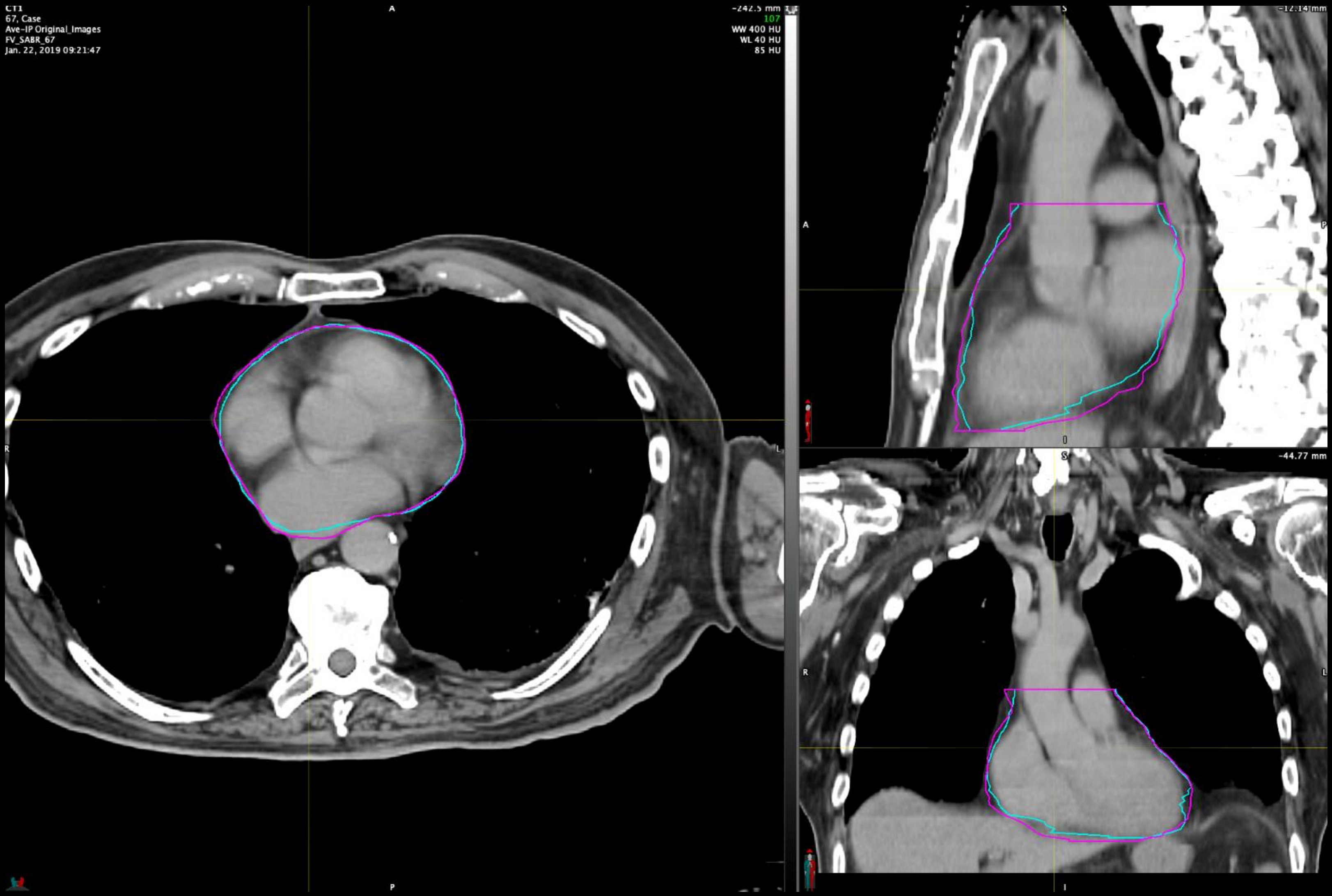

Heart – 95% HD 4.56mm, DSC 0.98

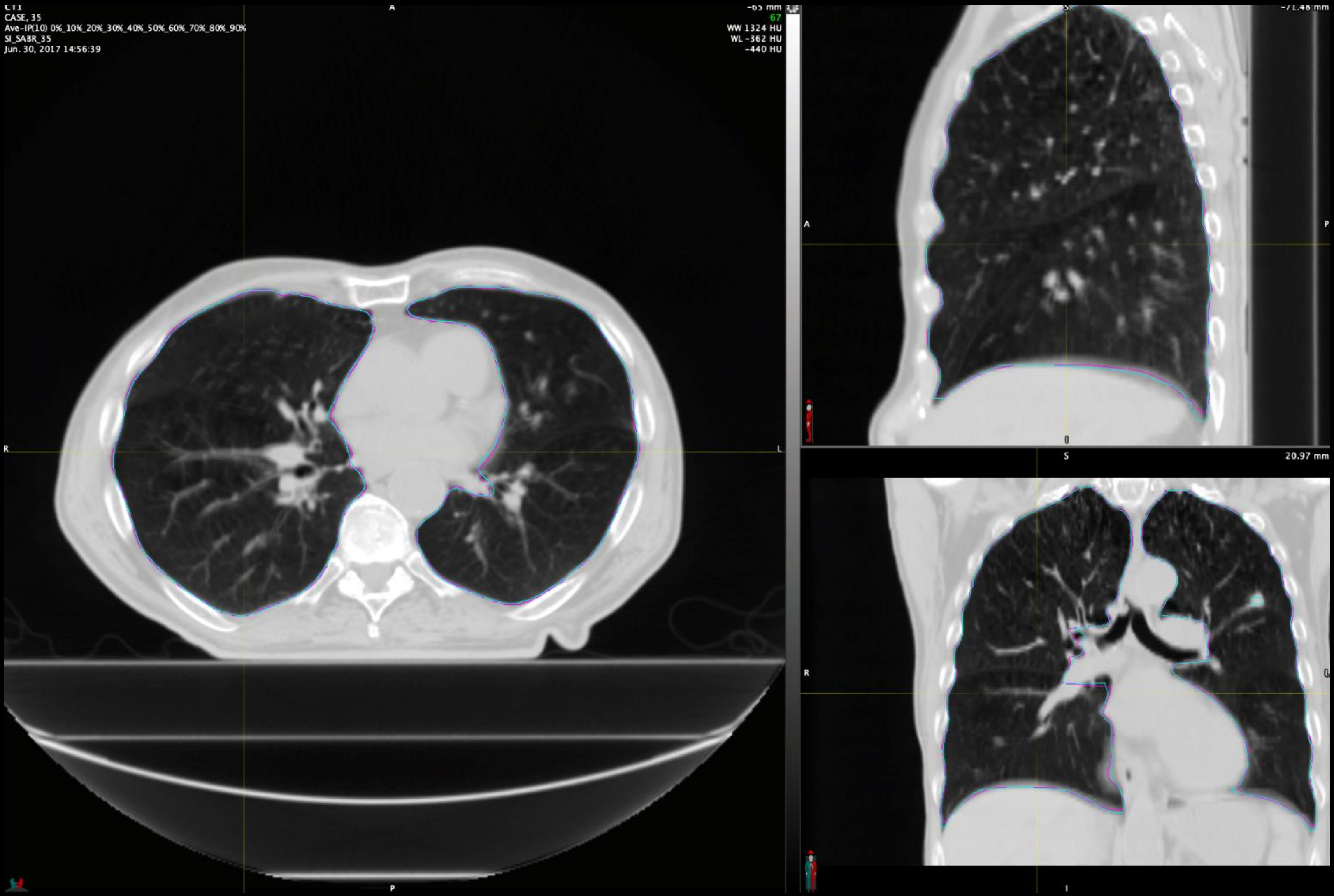

Lung left (lung window) – 95% HD 1.97mm, DSC 0.99
Lung right (lung window) – 95% HD 2.58mm, DSC 0.99

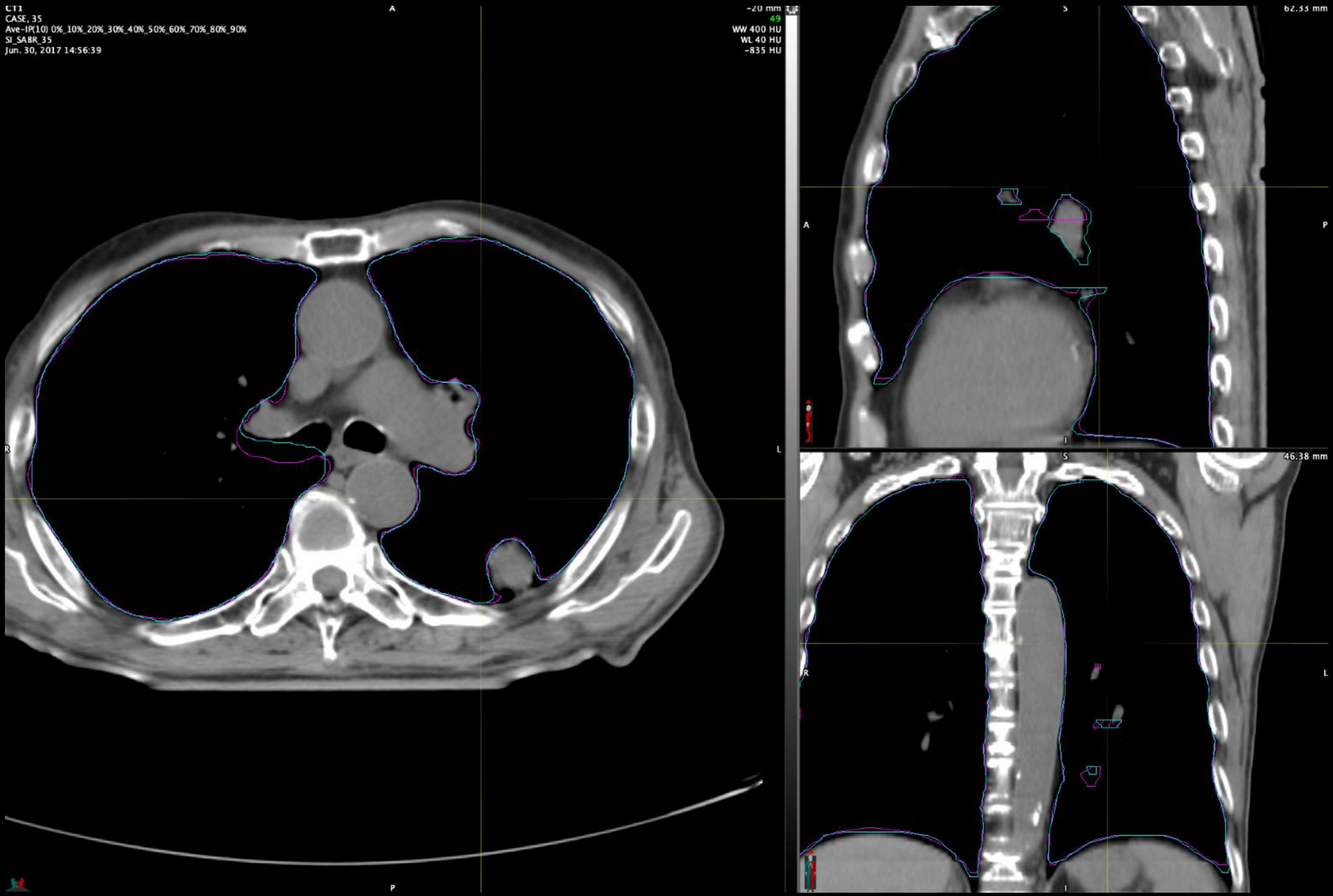

Lung left (soft tissue window) – 95% HD 1.97mm, DSC 0.99
Lung right (soft tissue window) – 95% HD 2.58mm, DSC 0.99

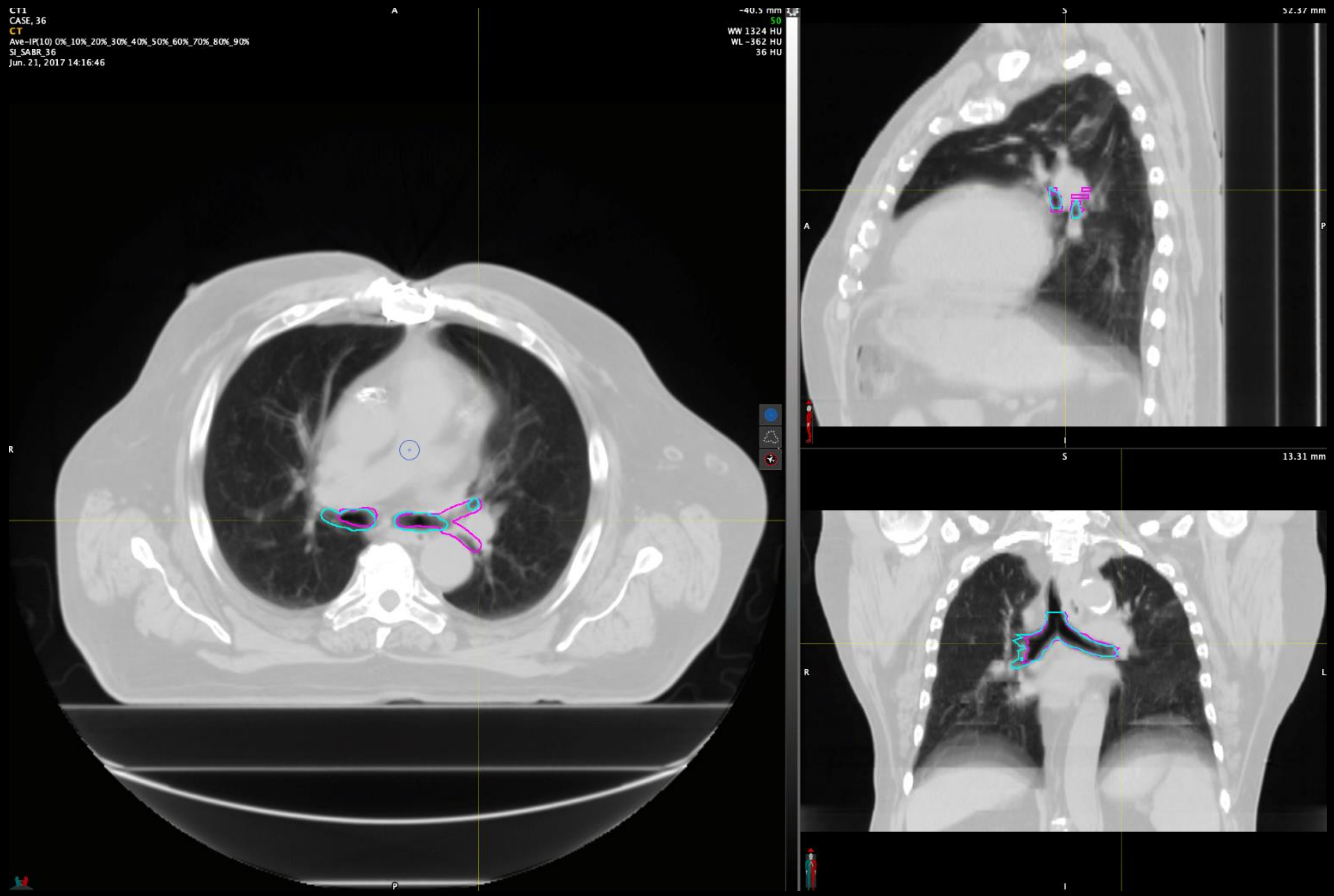

Proximal Bronchial Tree – 95% HD 7.13mm, DSC 0.65

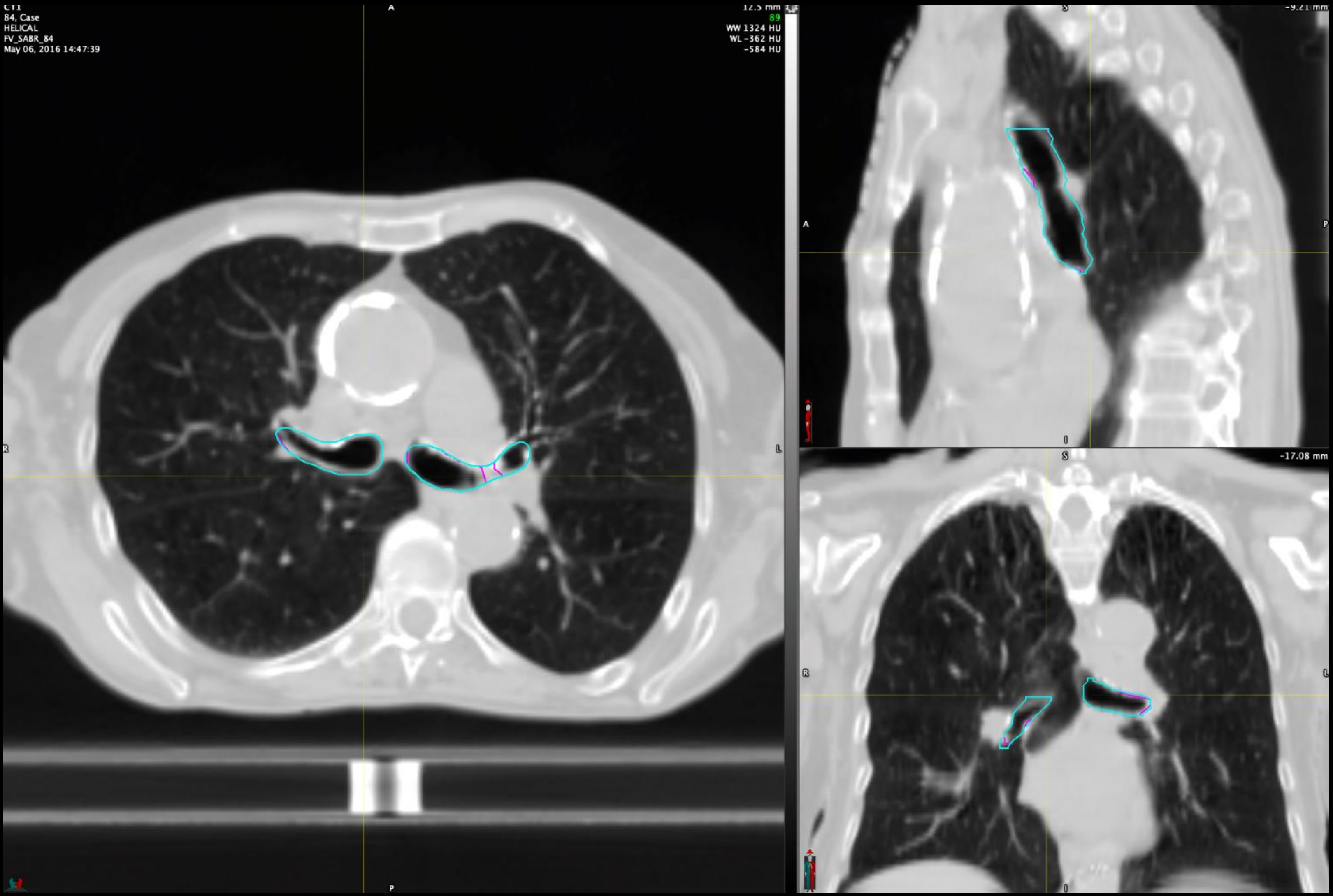

Proximal Bronchial Tree – 95% HD 1.73mm, DSC 0.97

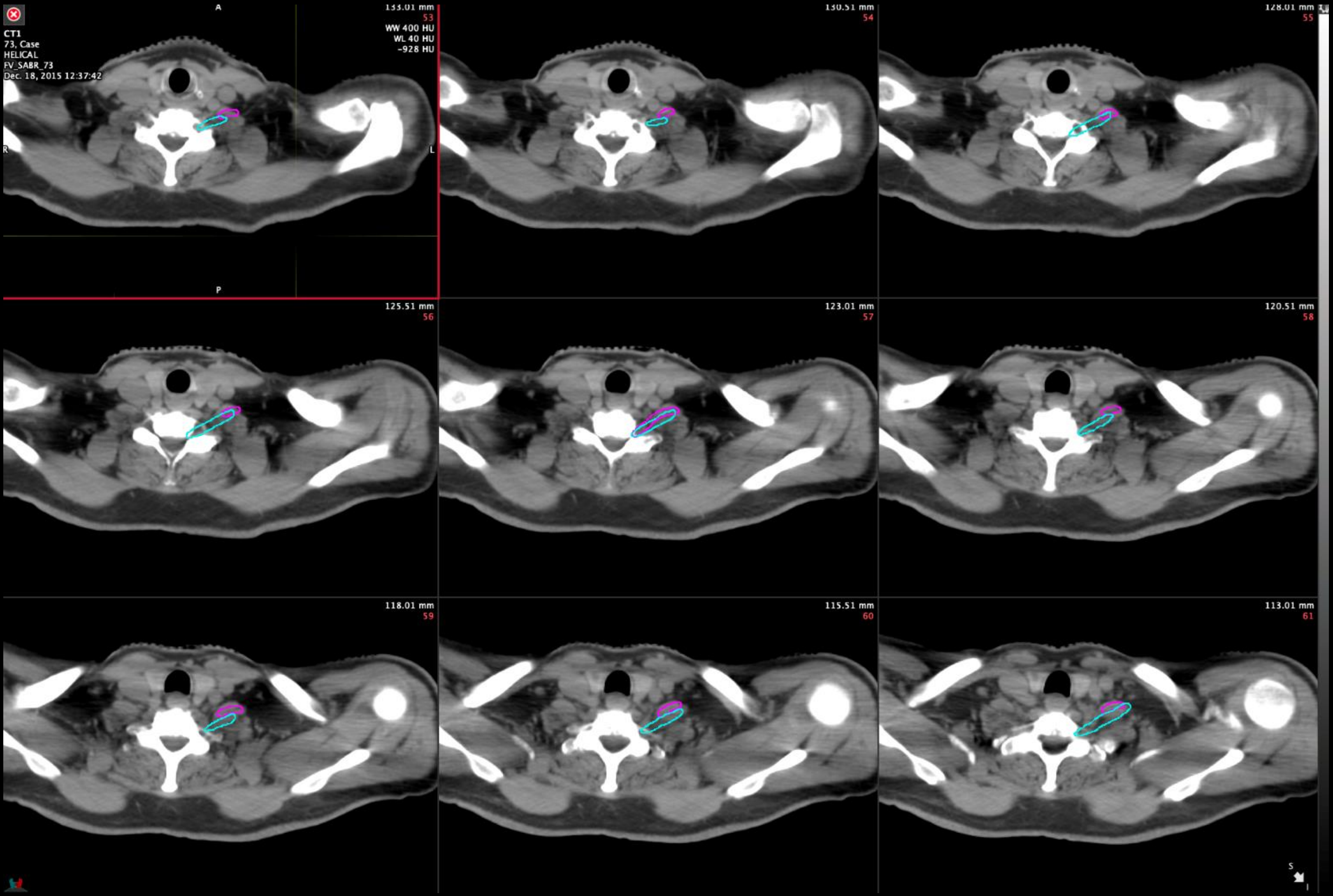

Brachial Plexus – 95% HD 13.65mm, DSC 0.19 (sup and inf borders cropped to same planes before comparison)

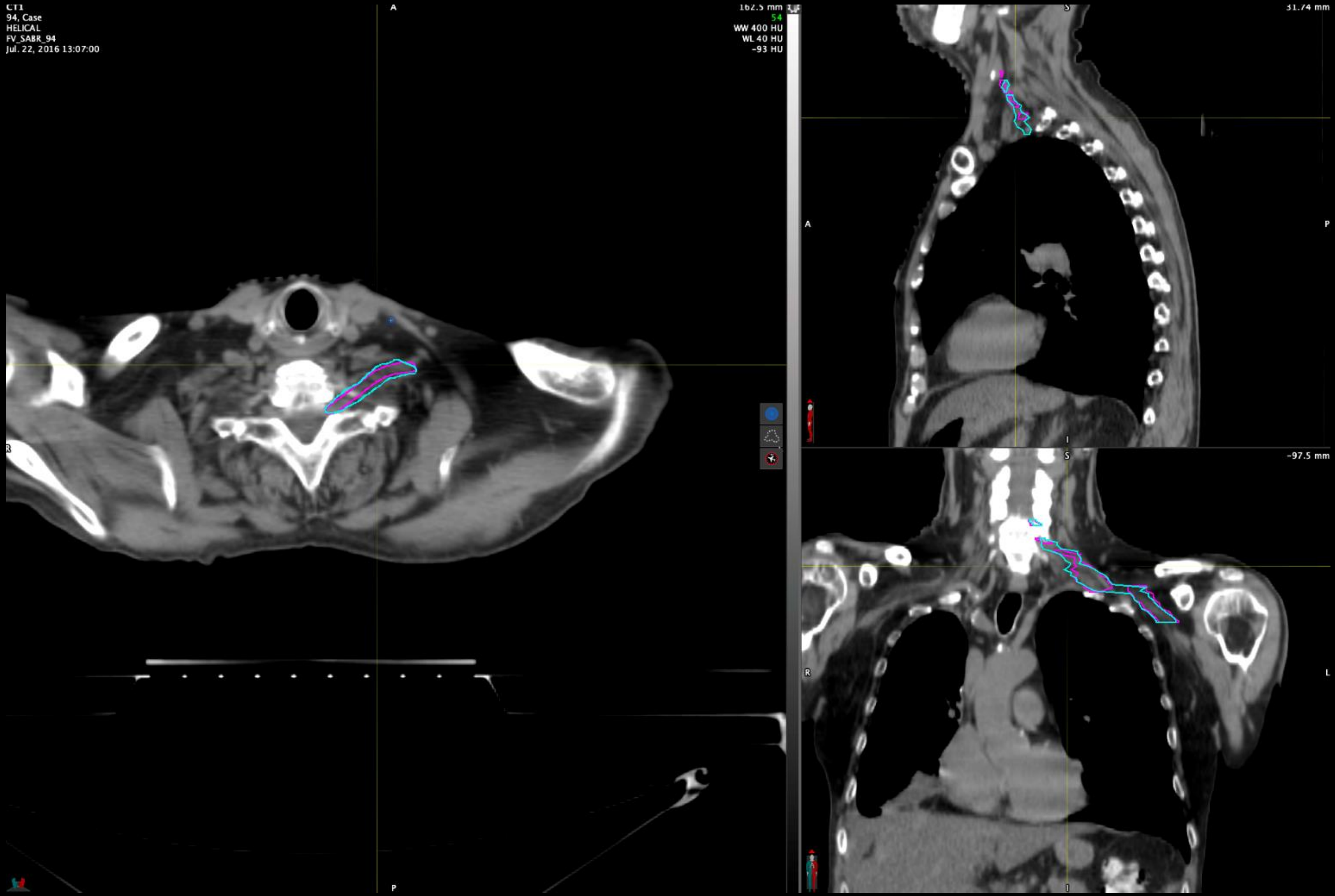

Brachial Plexus – 95% HD 2.99mm, DSC 0.81 (sup and inf borders cropped to same planes before comparison)

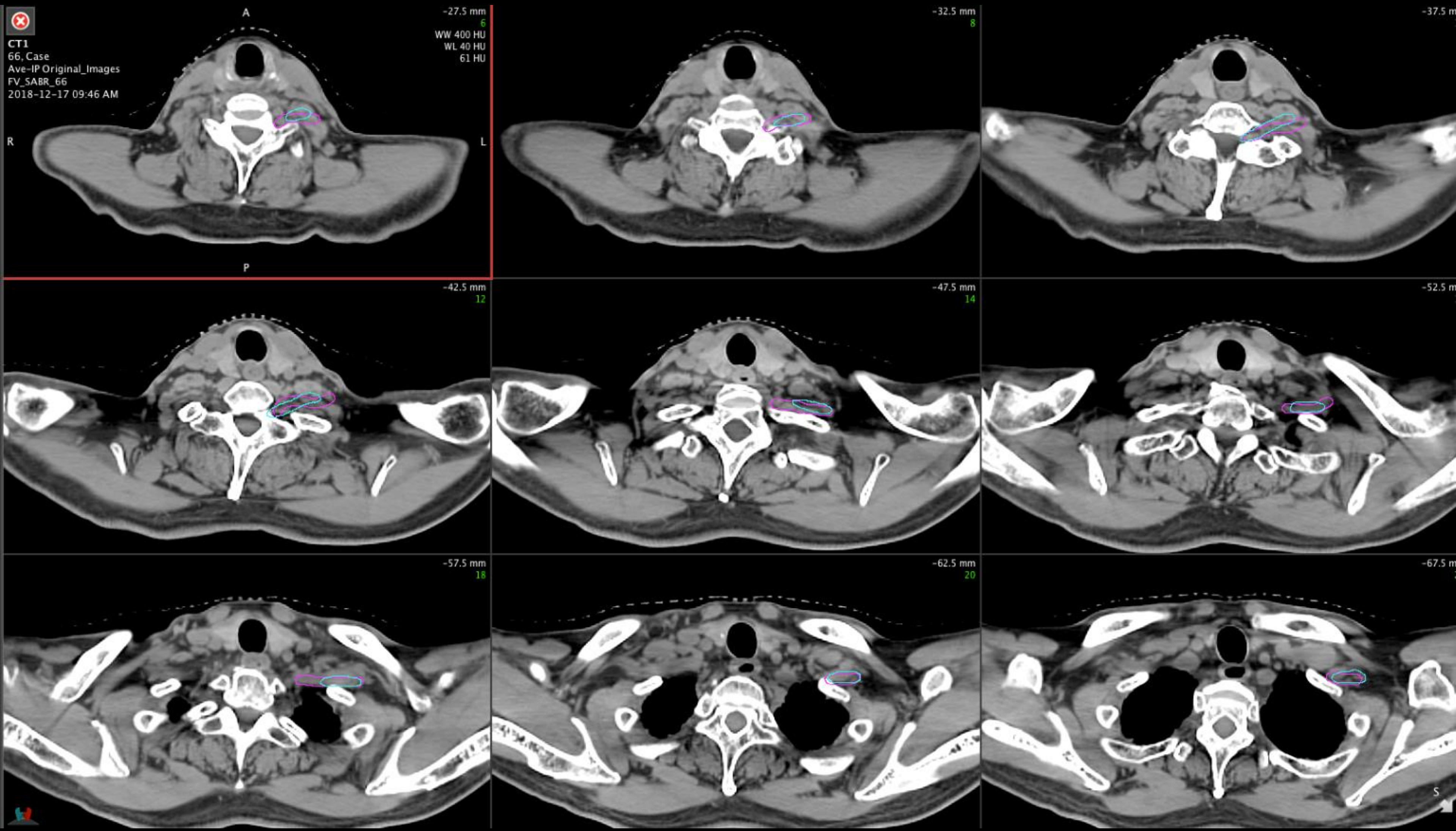

Brachial Plexus – 95% HD 6.04mm, DSC 0.53 (sup and inf borders cropped to same planes before comparison)

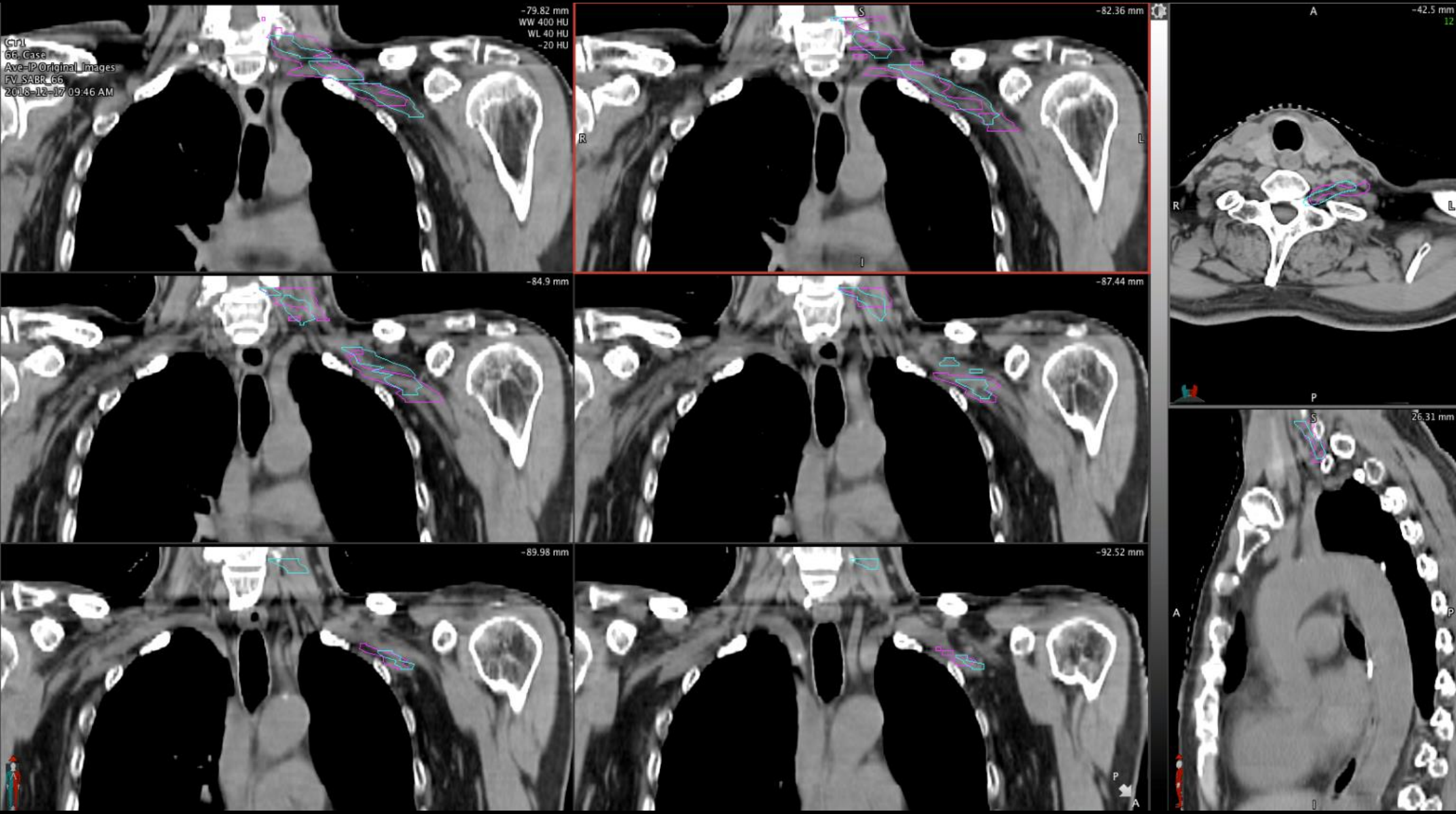

Brachial Plexus – 95% HD 6.04mm, DSC 0.53 (sup and inf borders cropped to same planes before comparison)

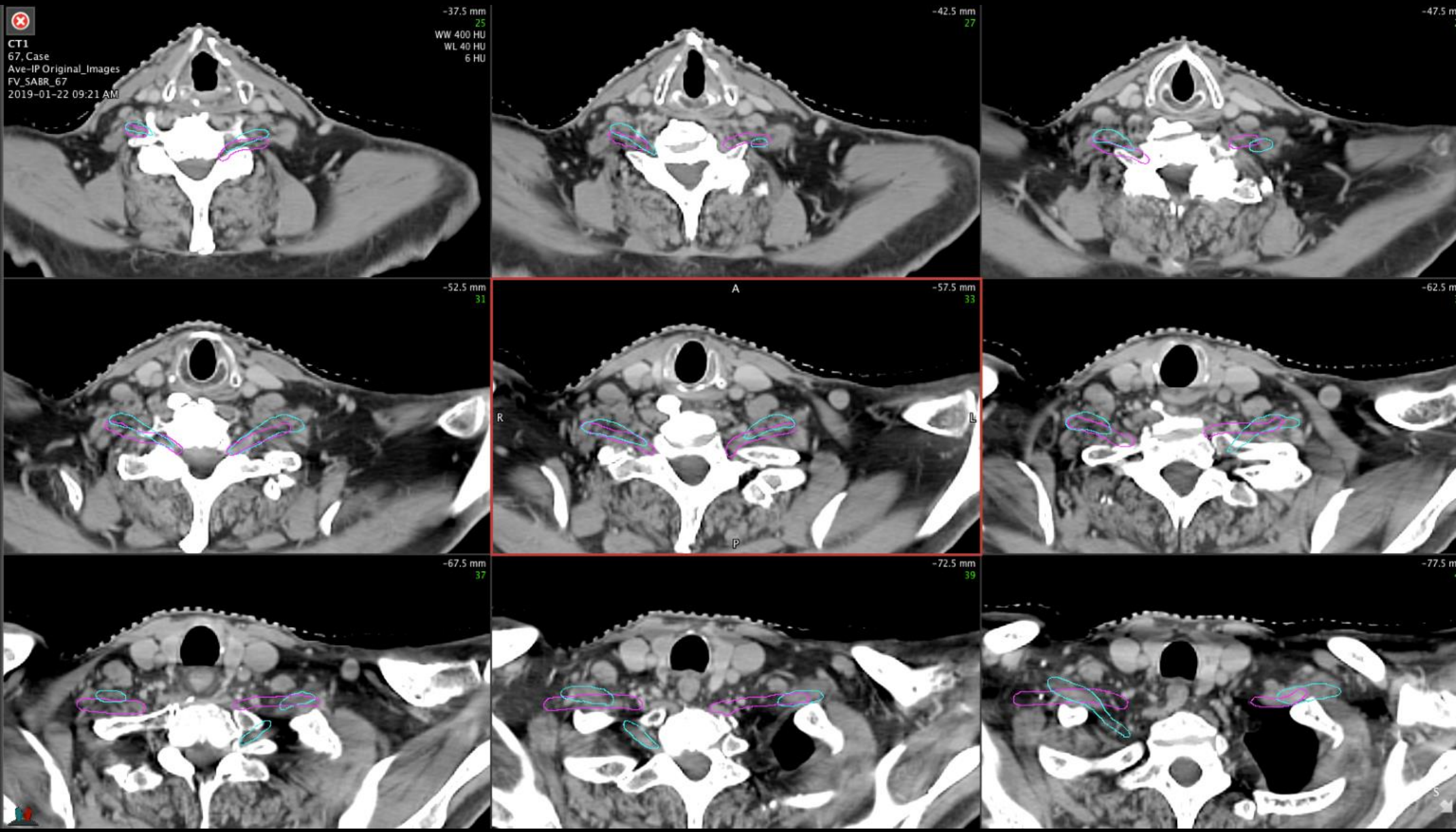

Brachial Plexus left – 95% HD 10.25mm, DSC 0.43 (sup and inf borders cropped to same planes before comparison)
Brachial Plexus right – 95% HD 10.43mm, DSC 0.39 (sup and inf borders cropped to same planes before comparison)

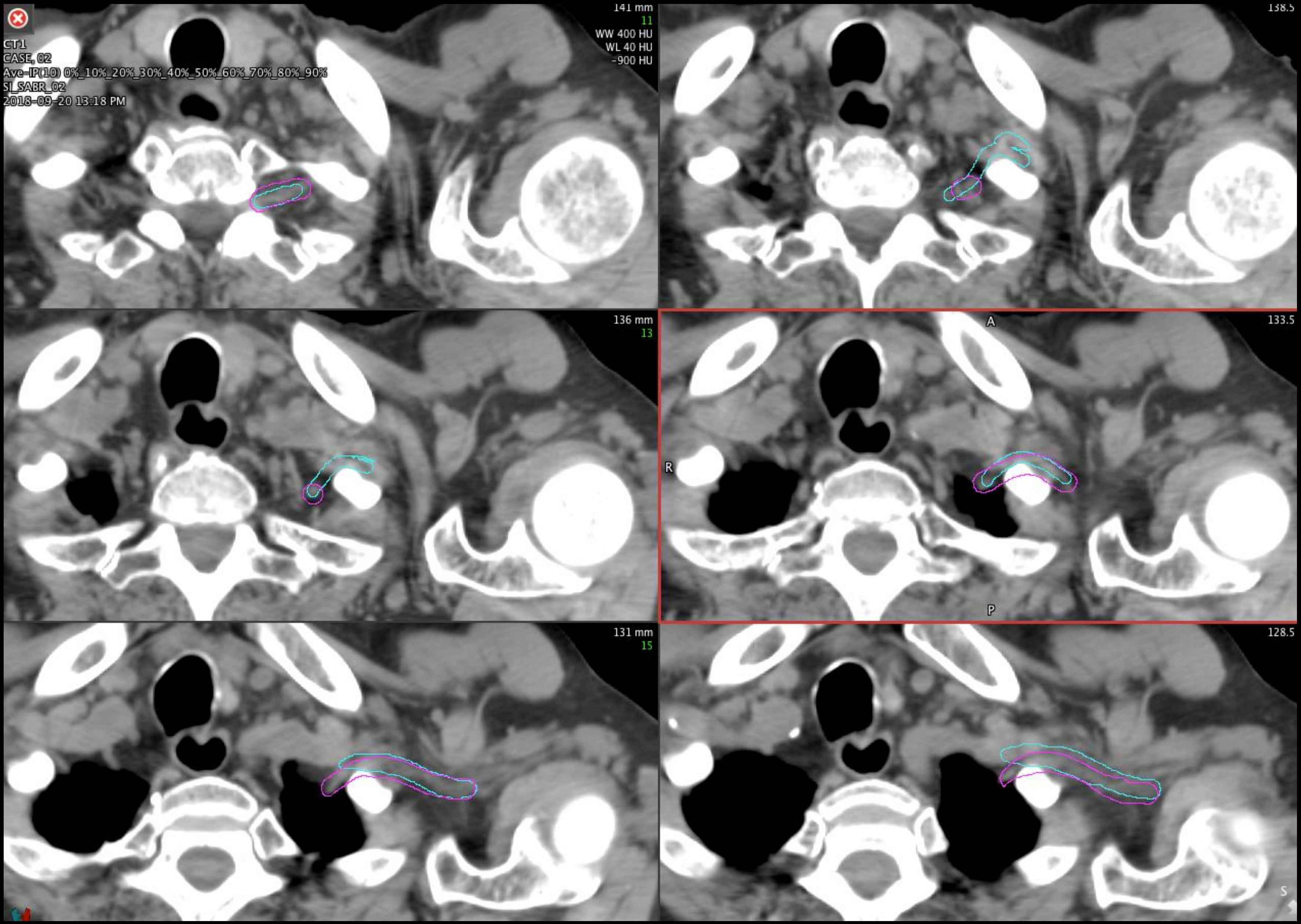

Brachial Plexus – 95% HD 4.45mm, DSC 0.68 (sup and inf borders cropped to same planes before comparison)

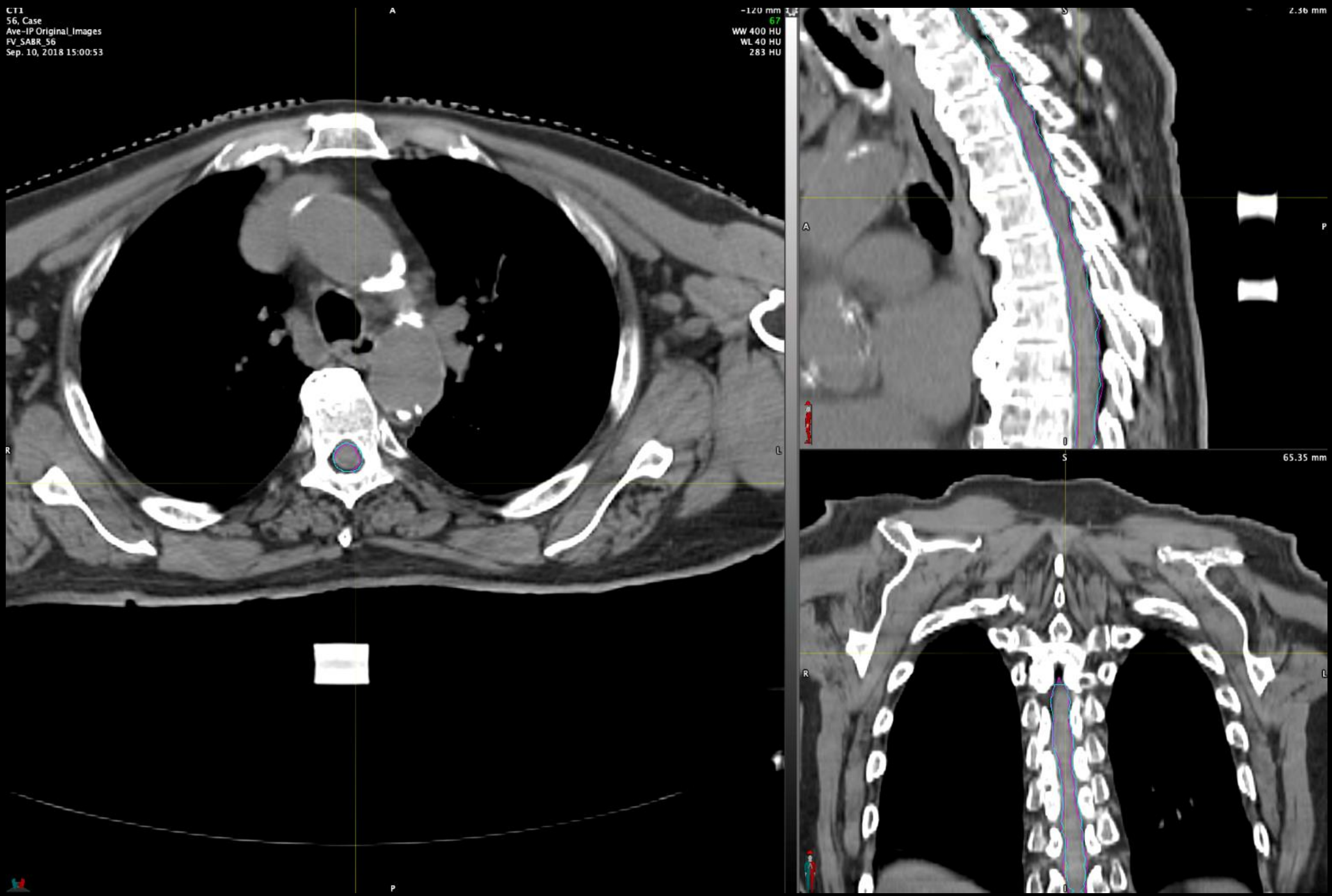

Spinal Cord – 95% HD 2.10mm, DSC 0.74 (sup and inf borders cropped to same planes before comparison)

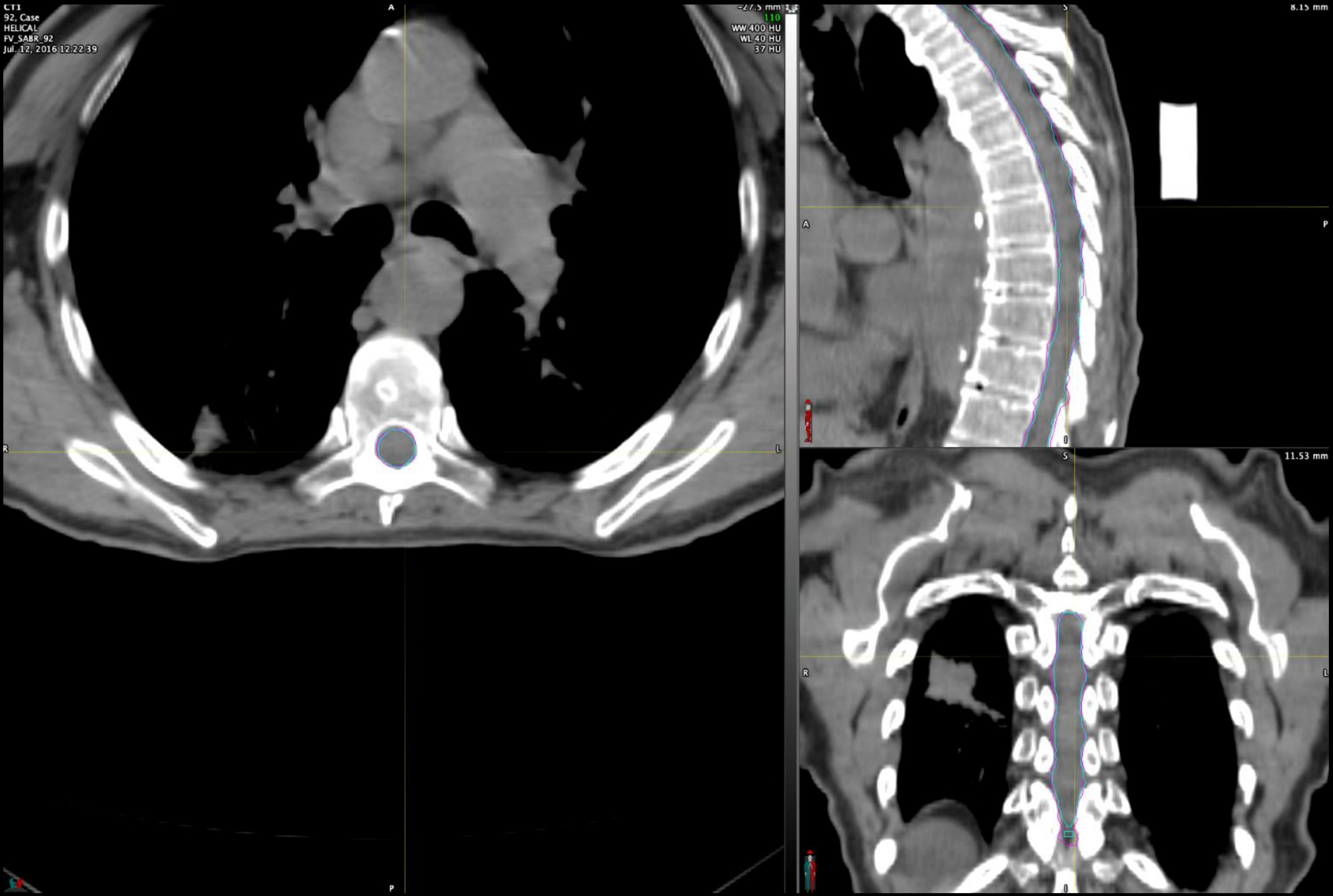

Spinal Cord – 95% HD 0.64mm, DSC 0.98 (sup and inf borders cropped to same planes before comparison)

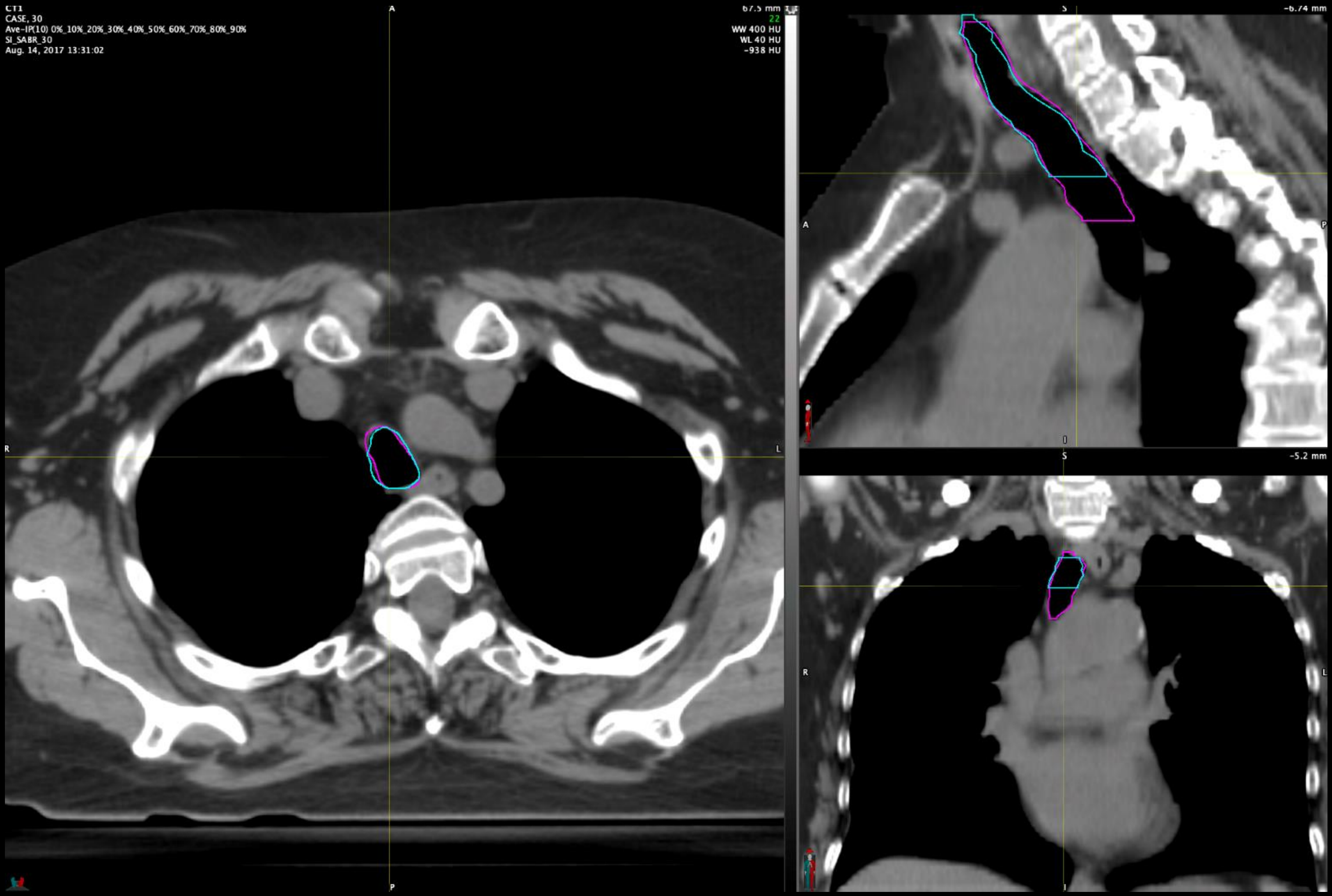

Trachea – 95% HD 7.85mm, DSC 0.79 (inf borders cropped to same planes before comparison)

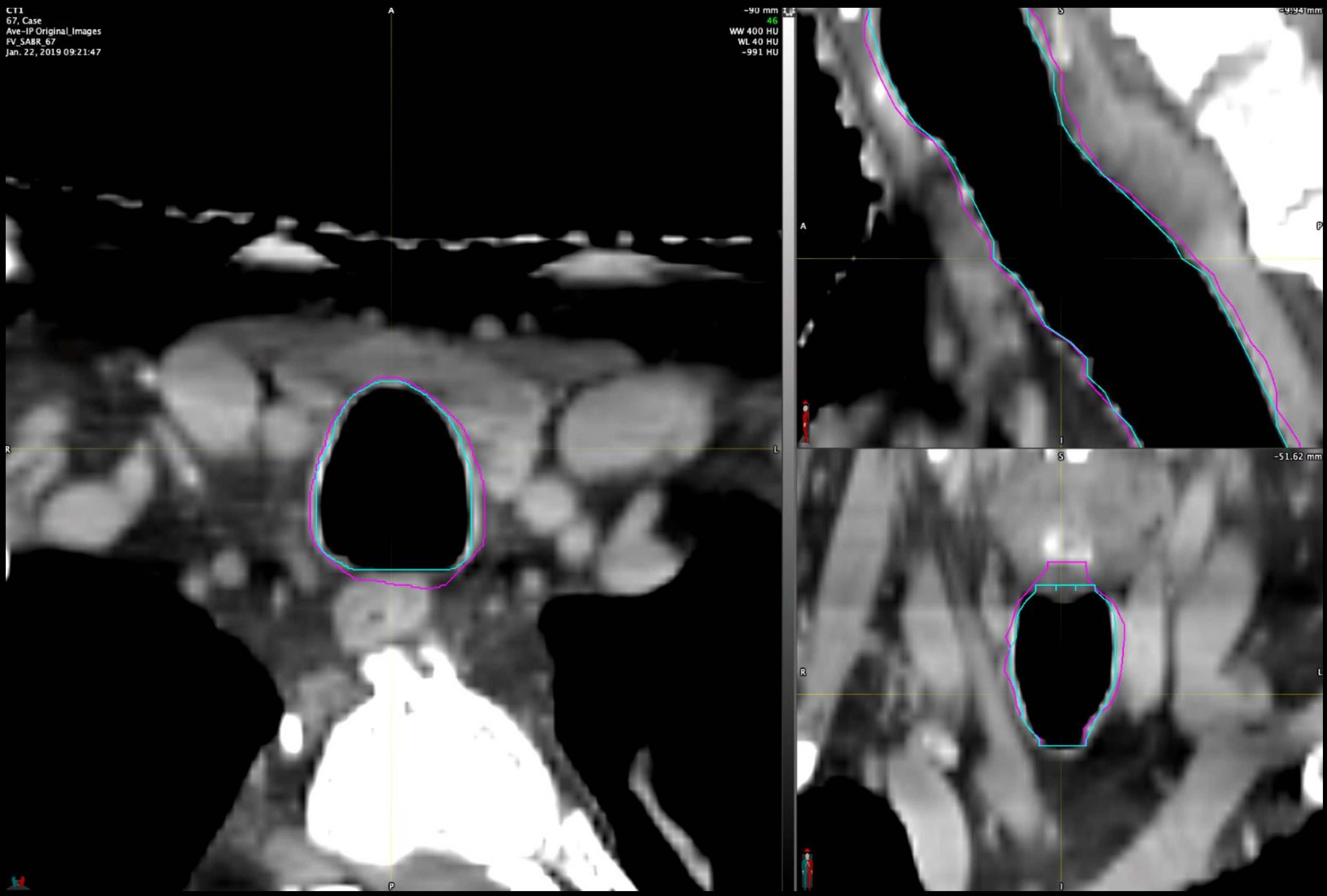

Trachea – 95% HD 3.25mm, DSC 0.98 (inf borders cropped to same planes before comparison)
