# Supplementary material for: Training and Validation of Deep Learning-Based Auto-Segmentation Models for Lung Stereotactic Ablative Radiotherapy Using Retrospective Radiotherapy Planning Contours
Source: Front Oncol. 2021 Jun 7;11:626499. doi: 10.3389/fonc.2021.626499 (PMC8215371; doi:10.3389/fonc.2021.626499)

Table S1. Summary of Dice Similarity Coefficient (DSC) and 95% Hausdorff distance (HD) metrics from comparing deep learning-based auto-segmented contours to clinical contours for lung stereotactic ablative radiotherapy planning on center A free-breathing and average 4D computed tomography image sets. (N - Number of validation contours used; GTV – gross tumor volume)

| **Structure** | **Center A FB N** | **Center A FB Mean DSC (range)** | **Center A FB Mean 95% HD (range; mm)** | **Center A AVG N** | **Center A AVG Mean DSC (range)** | **Center A AVG Mean 95% HD (range; mm)** |
| --- | --- | --- | --- | --- | --- | --- |
| Aorta | 20 | 0.94 (0.89-0.98) | 2.56 (1.56-4.16) | 18 | 0.92 (0.89-0.94) | 3.00 (2.53-3.93) |
| Esophagus | 28 | 0.80 (0.74-0.89) | 3.24 (2.05-5.05) | 21 | 0.80 (0.64-0.91) | 3.53 (2.21-6.94) |
| Heart | 28 | 0.96 (0.94-0.97) | 4.70 (2.95-5.83) | 22 | 0.95 (0.92-0.98) | 5.37 (3.25-10.9) |
| Lung Left | 28 | 0.98 (0.96-0.98) | 3.25 (2.15-17.27) | 15 | 0.97 (0.92-0.99) | 3.34 (2.36-6.73) |
| Lung Right | 28 | 0.97 (0.96-0.98) | 3.15 (2.61-4.74) | 17 | 0.98 (0.96-0.99) | 2.96 (2.55-4.48) |
| Brachial Plexus | 50 | 0.51 (0.04-0.81) | 6.85 (2.74-15.82) | 20 | 0.55 (0.33-0.80) | 6.75 (2.59-11.34) |
| Proximal Bronchial Tree | 49 | 0.85 (0.78-0.97) | 3.32 (1.73-5.39) | 22 | 0.82 (0.67-0.88) | 4.00 (2.99-5.61) |
| Spinal Cord | 28 | 0.91 (0.87-0.98) | 1.41 (0.56-2.01) | 22 | 0.90 (0.74-0.94) | 1.68 (1.31-2.69) |
| Trachea | 28 | 0.92 (0.83-0.95) | 2.26 (1.51-2.99) | 22 | 0.93 (0.89-0.98) | 2.27 (1.09-3.25) |
| GTV | 22 | 0.75 (0.57-0.89) | 5.03 (2.96-9.23) | 20 | 0.77 (0.67-0.90) | 5.02 (3.25-15.17) |

 Figure S1. Dice Similarity Coefficient (DSC, A) and 95% Hausdorff distance (HD, B) box plots from comparing deep learning-based auto-segmented contours to clinical contours for lung stereotactic ablative radiotherapy planning structures on center A free-breathing (FB) and average (AVG) 4D computed tomography image sets. (PBT – proximal bronchial tree; GTV – gross tumor volume).


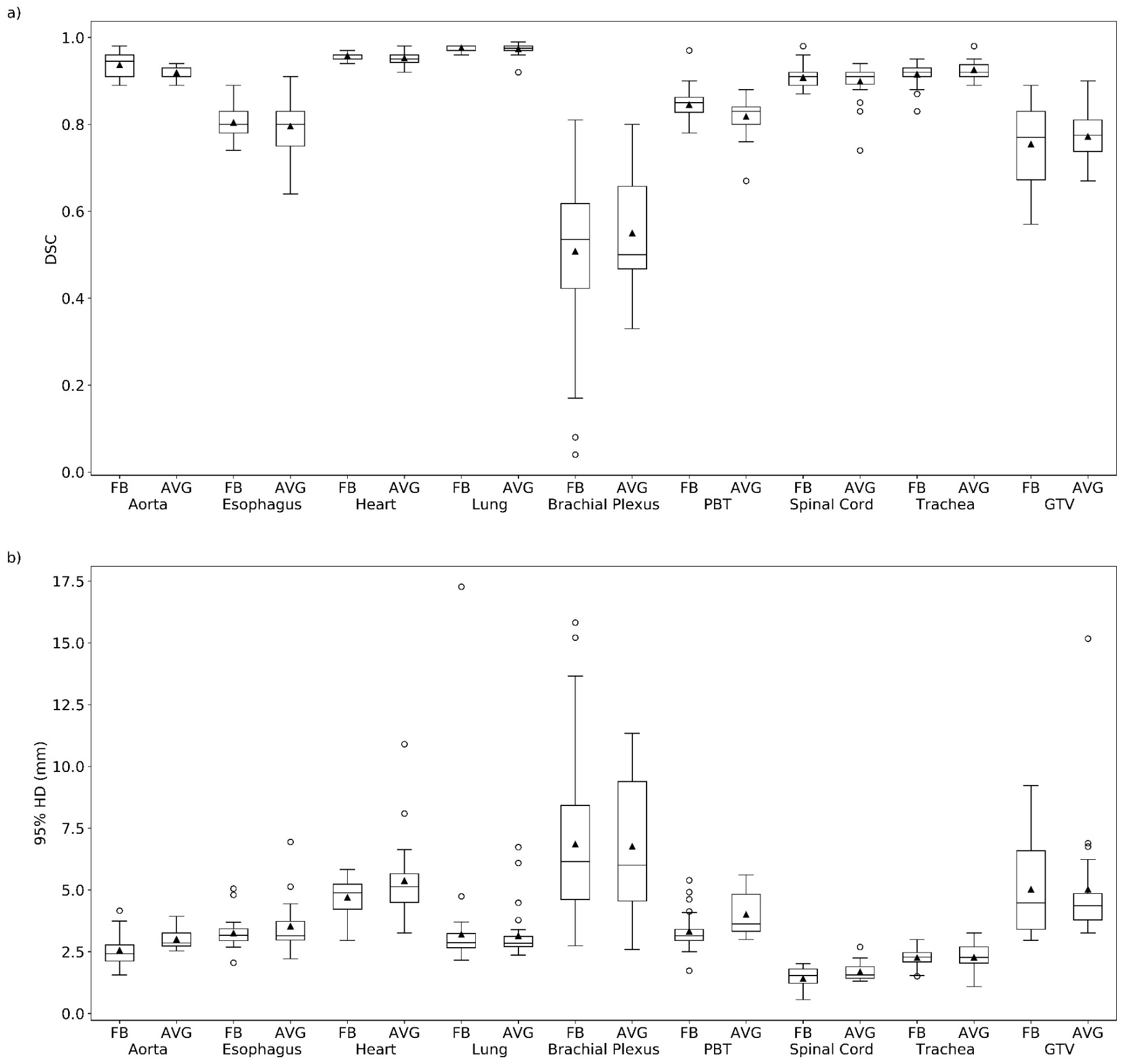

Supplement: Supplementary file 3 [file Table_1.docx]
